# Artificial intelligence and machine learning for magnetic nanoparticles and nanocarriers in cancer imaging, drug delivery, and magnetic control: a systematic review

**DOI:** 10.3762/bjnano.17.98

**Published:** 2026-10-20

**Authors:** Yier Yao, Wanis Nafo

**Affiliations:** 1 Academy of Life and Natural Sciences, Xi’an Jiaotong Liverpool University, 215123, Suzhou, China

**Keywords:** artificial intelligence, cancer drug delivery, machine learning, magnetic nanoparticles, MRI-visible nanocarriers

## Abstract

We reviewed artificial intelligence and machine learning in magnetic resonance imaging-visible magnetic nanoparticles, nanocarriers, and systems for imaging, drug delivery, hyperthermia, targeting, transport, and control. Using “preferred reporting items for systematic reviews and meta-analyses (PRISMA) 2020” methods, 2,283 records from seven databases yielded 65 studies. Predicted outcomes were colloidal stability, toxicity, uptake, magnetic descriptors, specific absorption rate, relaxivity, MRI signal, superparamagnetic iron oxide nanoparticle (SPION) quantification, drug release, biodistribution, and magnetic navigation. Evidence was strongest in bounded tasks: magnetic-property and relaxivity prediction, image-derived SPION mapping, hyperthermia modelling, and controlled magnetic transport. Most studies optimised isolated endpoints rather than integrating stability, MRI visibility, drug loading, release, tumour delivery, safety, and therapeutic response. External validation across datasets, laboratories, particle batches, biological models, scanners, and patient cohorts remains limited.

## Review

### 1 Introduction

Magnetic nanoparticles and nanocarriers are distinct in nanomedicine because their physicochemical properties can be coupled to diagnostic imaging, magnetic actuation, externally guided transport, and therapy [1]. Iron oxide nanoparticles, especially superparamagnetic iron oxide nanoparticles (SPIONs), are widely studied as MRI-visible systems because their relaxation changes produce contrast weighted by longitudinal relaxation time (*T*_1_) or transverse relaxation time (*T*_2_). This response depends on particle size, composition, coating, aggregation state, concentration, and acquisition conditions [2]. These carriers may incorporate polymeric shells, targeting ligands, drug payloads, thermoresponsive matrices, or hybrid magnetic reservoirs for imaging, drug delivery, hyperthermia, cell tracking, graft monitoring, and image-guided therapeutic evaluation [3]. Performance is therefore governed by the magnetic core and by interacting structural, surface, environmental, magnetic, biological, and imaging variables.

Artificial intelligence and machine learning (AI/ML) methods now address this multivariable design space. They have been used to predict nanoparticle size, zeta potential, cytotoxicity, cellular uptake, magnetic descriptors, specific absorption rate, relaxivity, and MRI signal behaviour [4]. Image analysis models have segmented or quantified SPION-labelled cells, tumour-associated probe accumulation, magnetic particle imaging signal, and image-derived markers of therapeutic response [5]. Optimisation and control algorithms have examined formulation conditions, synthesis parameters, magnetic-field inputs, particle trajectories, swarm behaviour, and externally guided delivery [6]. These applications include regression, support vector machines, ensemble methods, neural networks, reinforcement learning, physics-informed models, surrogate models, and nanoparticle quantitative structure–activity relationship (nano-QSAR) and nanoparticle quantitative structure–property relationship (nano-QSPR) frameworks [7]. They seek predictive structure in datasets where one-variable analysis cannot represent interactions among material, biological, and imaging factors.

The principal AI/ML methods used in this literature differ in how they learn relationships between inputs and outputs. Artificial neural networks (ANNs) use interconnected layers of weighted nodes to approximate nonlinear relationships and are commonly applied to regression or classification problems. Random forests combine predictions from multiple decision trees and are suited to structured tabular data, with the additional advantage of providing measures of feature importance. Support vector machines (SVMs) identify decision boundaries that maximise separation between classes, or, in support vector regression, fit continuous outcomes within a defined error margin; kernel functions allow nonlinear relationships to be represented. Convolutional neural networks (CNNs) learn spatial features directly from images and are therefore particularly useful for segmentation, detection, and quantitative analysis of MRI or microscopy data. Reinforcement learning differs from these supervised approaches because an agent learns a sequential decision policy through rewards and penalties generated by its interaction with an environment, making it applicable to magnetic navigation and control problems. These distinctions are relevant because algorithm suitability depends on the endpoint, data structure, sample size, and validation design.

The application of AI/ML to magnetic nanoparticle research has expanded substantially over the past decade. Early studies from 2015–2019 applied methods such as SVMs and data-driven regression to SPION quantification, cell tracking, and nanoparticle toxicity. From 2020–2022, applications broadened to nano-QSAR modelling, deep-learning-based SPION quantification, and prediction of nanoparticle biological and magnetic behaviour. From 2023–2026, the field expanded further through larger curated datasets, ensemble learning, synthesis optimisation, reinforcement learning, physics-informed models, and AI-assisted magnetic navigation and control. This progression reflects a shift from isolated prediction and classification tasks toward models addressing multiple material, imaging, biological, and delivery variables.

The literature remains fragmented. Some studies focus on physicochemical stability, aggregation, surface chemistry, or toxicity, whereas others address magnetic properties, hyperthermia, relaxivity, SPION quantification, magnetic navigation, transport under flow, or drug release from magnetic reservoirs [8]. Data sources range from narrow experimental systems to curated cross study datasets, simulated outputs, phantom imaging, animal models, and clinical imaging cohorts [9]. This heterogeneity obscures which AI/ML methods have been applied to MRI-visible magnetic nanoparticles and nanocarriers, which endpoints dominate, and where methodological weaknesses remain. High internal accuracy also may not transfer across particle batches, biological media, scanners, pulse sequences, laboratories, tumour models, or patient populations [10].

A systematic review is therefore needed to consolidate the evidence by material system, modelling strategy, endpoint, descriptor design, and validation practice. Existing reviews cover AI/ML in nanotoxicology, nanoparticle design, medical imaging, magnetic hyperthermia, and drug delivery, but these areas are usually treated separately. Previous reviews have addressed narrower areas, including AI in magnetic hyperthermia [11], ML-assisted magnetic nanoparticles for central nervous system applications [12], and ML-guided nanoparticle design for cancer drug delivery [13]. The present review extends beyond these application-specific perspectives by integrating stability, toxicity, magnetic properties, MRI performance, SPION quantification, drug delivery, and magnetic control within one systematic synthesis. It further compares model types, descriptor selection, data sources, validation approaches, and translational relevance across these domains. For MRI-visible magnetic nanocarriers, that separation is limiting because translational performance requires multiple functions within one platform [14]. Similarly, image-based quantification of SPION-labelled cells does not inform formulation design unless imaging descriptors are linked to material and biological variables [12–13]. Focused synthesis is needed to determine whether current models support integrated nanocarrier design or remain limited to isolated prediction tasks.

This review evaluates studies applying AI/ML methods to MRI-visible magnetic nanoparticles, magnetic nanocarriers, and related magnetic nanosystems for imaging, drug delivery, hyperthermia, targeting, transport, or externally controlled delivery. It maps nanoparticle systems, methods, descriptors, endpoints, imaging and magnetic performance, diagnostic and therapeutic integration, and validation practices affecting model interpretation. Following PRISMA 2020 guidelines, the review includes 76 studies selected from 2,283 records identified across the Association for Computing Machinery (ACM) Digital Library, Excerpta Medica Database (EMBASE), Institute of Electrical and Electronics Engineers (IEEE) Xplore, PubMed, ScienceDirect, Scopus, and Web of Science.

The Methods section describes the search strategy, eligibility criteria, screening process, and synthesis approach. The Results section examines AI/ML applications in physicochemical stability, magnetic descriptors, MRI performance, integrated drug-delivery design, transport, magnetic navigation, and evidence quality. The Conclusion interprets descriptor engineering, generalisability, validation limitations, and requirements for clinically relevant AI/ML-assisted design. The manuscript concludes by identifying priorities for transparent preprocessing, external validation, multiobjective optimisation, and integration of stability, imaging, delivery, and therapeutic endpoints.

### 2 Methods

#### 2.1 Search strategy

We searched for studies applying AI/ML methods to magnetic nanoparticles, MRI-visible magnetic nanocarriers, and related magnetic nanosystems for drug delivery, imaging, targeting, hyperthermia, or externally controlled transport. The ten-year search used PubMed, Web of Science, Scopus, EMBASE, IEEE Xplore, ScienceDirect, and the ACM Digital Library to cover nanomedicine, materials science, biomedical imaging, engineering, and computer science. The search primarily targeted AI/ML studies, but experimentally based magnetic nanoparticle studies were retained when they were necessary to interpret AI/ML findings on MRI contrast, colloidal stability, magnetic behaviour, coating effects, toxicity, or nanocarrier function.

Search strings combined magnetic nanoparticle, AI/ML, and application terms. Magnetic nanoparticle terms were “magnetic nanoparticle”, “magnetic nanocarrier”, “iron oxide nanoparticle”, “superparamagnetic iron oxide nanoparticle”, “SPION”, “Fe_3_O_4_”, “Fe_2_O_3_”, “ferrite”, “magnetic nanogel”, “magnetic hydrogel”, “magnetic ferrogel”, “magnetic scaffold”, “magnetic contrast agent”, and “MRI-visible nanoparticle”. AI/ML terms were “artificial intelligence”, “machine learning”, “deep learning”, “neural network”, “artificial neural network”, “convolutional neural network”, “support vector machine”, “support vector regression”, “random forest”, “gradient boosting”, “extreme gradient boosting (XGBoost)”, “Bayesian optimisation”, “genetic algorithm”, “reinforcement learning”, “physics-informed neural network”, “surrogate model”, “predictive modelling”, “data-driven modelling”, “quantitative structure–property relationship (QSPR)”, “quantitative structure–activity relationship (QSAR)”, “nano-QSPR”, and “nano-QSAR”. Application terms were “magnetic resonance imaging”, “MRI”, “relaxivity”, “MRI contrast”, “magnetic particle imaging (MPI)”, “SPION quantification”, “cell tracking”, “graft tracking”, “hyperthermia”, “drug delivery”, “drug release”, “biodistribution”, “cellular uptake”, “toxicity”, “tumour accumulation”, “magnetic targeting”, “magnetic navigation”, “magnetic actuation”, “swarm control”, “image-guided delivery”, “closed-loop delivery”, and “robotic delivery”. Terms were combined with Boolean operators and adapted to each database.

**Figure 1:**
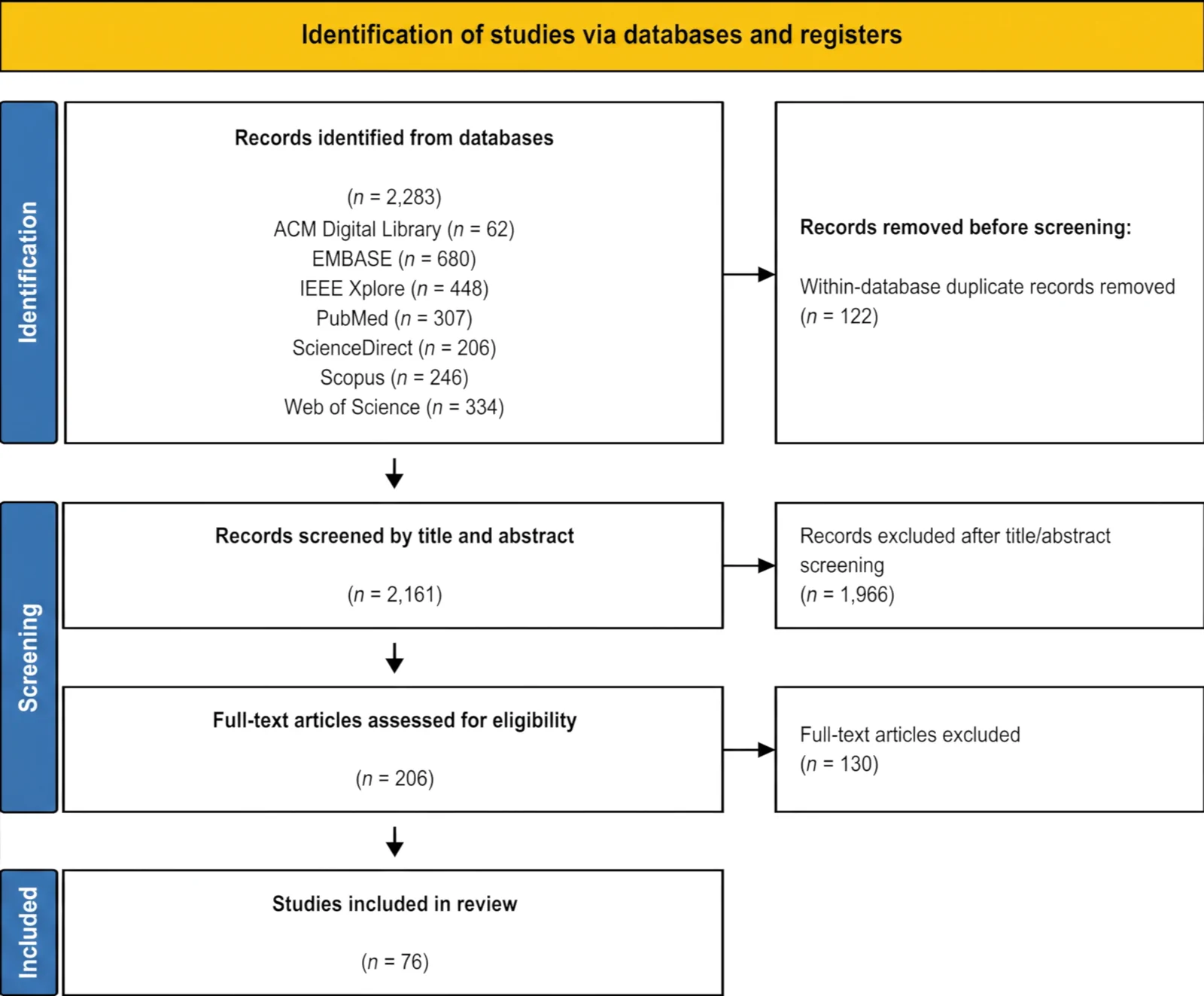
PRISMA flow diagram for systematic review depicting phases of identification of studies.

The search identified 2,283 records, that is, 62 from the ACM Digital Library, 680 from EMBASE, 448 from IEEE Xplore, 307 from PubMed, 206 from ScienceDirect, 246 from Scopus, and 334 from Web of Science. Within-database deduplication removed 122 duplicates, leaving 2,161 records for title and abstract screening. Preliminary screening retained 195 articles for full-text assessment. Full-text review excluded 130 articles, leaving 76 included studies. Figure 1 summarises the study selection.

#### 2.2 Eligibility criteria

Studies had to fall within the ten-year window. Two earlier studies were retained as legacy and benchmark evidence for included studies, namely, early SVM-based MRI quantification of SPION-labelled cells, and foundational MRI contrast-agent behaviour in magnetic nanoparticle systems. MRI-visible systems were magnetic materials commonly used or measurable as MRI or MPI contrast agents. Magnetic systems were eligible without direct MRI testing when the system, endpoint, or task was relevant to MRI-visible magnetic nanocarrier development. For the primary AI/ML synthesis, studies had to apply AI/ML for prediction, classification, optimisation, segmentation, quantification, control, or design. Experimental or mechanistic magnetic nanoparticle studies without AI/ML were eligible only as supporting evidence when they directly clarified interpretation of the AI/ML findings. Eligible approaches included regression and classification models, support vector machines, random forests, gradient boosting, artificial neural networks, deep learning, convolutional neural networks, reinforcement learning, physics-informed neural networks, genetic algorithms, Bayesian optimisation, surrogate modelling, QSPR, QSAR, nano-QSPR, nano-QSAR, and related data-driven methods with identifiable inputs, outputs, and performance metrics. Studies trained on simulation or computational fluid dynamics outputs were included when a learning algorithm predicted particle transport, flow, heating, imaging, control, or delivery behaviour.

The endpoint had to relate to MRI-visible magnetic nanocarrier development, diagnostic performance, or therapeutic function. Eligible endpoints included colloidal stability, aggregation, hydrodynamic size, polydispersity index, zeta potential, coating behaviour, synthesis conditions, saturation magnetisation, coercivity, remanence, exchange bias, specific absorption rate, MRI relaxivity, MRI signal output, SPION concentration mapping, MPI signal, image-derived magnetic biomarkers, cellular uptake, cytotoxicity, biodistribution, pharmacokinetics, tumour accumulation, drug loading, drug release, magnetic targeting, magnetic navigation, magnetic actuation, transport in flow, swarm behaviour, and image-based localisation of magnetic carriers. Therapeutic relevance covered magnetic nanocarriers used for drug delivery, controlled release, magnetic targeting, hyperthermia, externally guided delivery, or magnetic control. Diagnostic relevance covered MRI-visible magnetic nanoparticles used for MRI contrast, relaxivity prediction, SPION quantification, MPI, cell or graft tracking, and other imaging-based assessments involving magnetic carriers in oncology.

Studies were excluded if they were outside the ten-year window, did not involve magnetic nanoparticles or MRI-visible magnetic nanosystems, investigated non-magnetic nanoparticles without magnetic-delivery, magnetic-control, MRI, or MPI relevance, used AI only for general tumour segmentation, radiomics, or image classification without magnetic nanoparticles, focused on small-molecule drug discovery without a nanocarrier component, reported synthesis or characterisation without AI/ML and without direct interpretive relevance to the reviewed AI/ML evidence, used conventional numerical simulation without a learning component, or lacked enough methodological detail to identify the nanoparticle composition, AI/ML model, endpoint, quantitative outcome, or performance metric. Reviews, editorials, opinion articles, patents, and abstracts without adequate technical detail were excluded. Conference papers were eligible when sufficient methodological and quantitative detail was available. Experimental or mechanistic papers without AI/ML were not counted as primary included studies, but were retained as supporting references when they clarified colloidal stability, coating behaviour, magnetic interactions, toxicity mechanisms, MRI contrast, or nanocarrier interpretation.

Screening used title and abstract review followed by full text assessment. Titles, abstracts, and full texts were screened independently by two reviewers, with disagreements resolved by discussion and full text rechecking. Data extraction was also performed by two reviewers, and uncertain entries were verified against the full text. Extracted items were nanoparticle system, AI/ML method, descriptors, endpoint, data source, validation approach, performance metrics, and relevance to MRI-visible magnetic nanocarrier design.

### 3. Results

#### 3.1 AI and ML models for stability, aggregation, and physicochemical properties

**3.1.1 Stability descriptors and biological response.** Colloidal stability is essential for MRI-visible magnetic nanocarriers because aggregation changes hydrodynamic size, effective surface area, cellular uptake, magnetic interactions, and biological exposure. Stability was represented mainly by hydrodynamic diameter, polydispersity index (PDI), zeta potential, coating chemistry, particle concentration, exposure duration, pH, ionic strength, and temperature. These variables functioned as prediction targets or inputs for toxicity, uptake, magnetic response, and process optimisation models.

Dynamic toxicity simulations identified concentration and exposure duration as dominant predictors of viability loss [15]. Exposure conditions outweighed static descriptors, so nanoparticle response could not be inferred from initial properties alone. A simulated annealing leave-one-out cross-validation generalised radial basis function (SA-LOOCV-GRBF) model used hydrodynamic diameter, concentration, incubation time, and surface charge to predict iron oxide nanoparticle cytotoxicity in kidney cells [16]. The model used 58 complete observations from six studies and showed maximum predicted viability at 67.5 nm, indicating a nonlinear, exposure-dependent relationship between hydrodynamic size and cell viability. Toxicity was therefore governed by particle size, dose, time, transport, and surface charge. Aggregation and pH-controlled surface charge modify the effective particle state presented to cells.

Surface chemistry was modelled with nano-QSAR and automated machine learning frameworks. In pancreatic cancer and endothelial cell models, nano-QSAR linked uptake of functionalised magneto-fluorescent nanoparticles to molecular weight, carbon percentage, topological polar surface area, ring count, and atom count of surface modifiers. Atom count was the strongest contributor in both models [17]. Two nano-QSAR models based on 109 surface-functionalised nanoparticles achieved coefficient of determination (*R*^2^) values of 0.822 for PaCa-2 cells and 0.885 for human umbilical vein endothelial cells (HUVECs). The higher HUVEC performance suggests that uptake predictability depends on the biological model. Automated nano-QSAR simulation of SPION-based MRI agents identified magnetic core type and particle size as major determinants of cell viability [18]. The logistic regression model achieved 100% validation accuracy and indicated a toxicity transition for maghemite-core SPIONs at approximately 15 nm. Size effects therefore cannot be interpreted independently of magnetic core composition. Quasi-simplified molecular-input line-entry system (quasi-SMILES) simulation encoded post-exposure time, dose, toxicity endpoint, and polyethylene glycol (PEG) coating status as symbolic descriptors for predicting metal oxide nanoparticle safety under different experimental conditions [19]. The classification model used 574 measurements across seven metal oxide nanoparticle types and achieved a validation accuracy of 0.986. Exposure context therefore improves nanoparticle safety classification.

Predictive performance across stability-related models varied with the endpoint and dataset. The nano-QSPR model for zeta potential achieved *R*^2^ = 0.912, with external predictive coefficients above 0.897 [4], whereas nano-QSAR models of nanoparticle uptake achieved *R*^2^ values of 0.822 and 0.885 for pancreatic and endothelial cells, respectively [17]. In SPION toxicity classification, logistic regression achieved 100% validation accuracy [18]; however, the dataset contained only 16 SPIONs, including five validation samples, limiting confidence in the apparent superiority of this model. Quasi-SMILES modelling achieved a validation accuracy of 0.986 across 574 metal-oxide measurements [19]. These results demonstrate strong performance for specific physicochemical and biological endpoints, but do not identify a generally superior algorithm for colloidal stability because aggregation and long-term stability were rarely evaluated as direct prediction endpoints.

AI/ML analysis of SPION surface chemistry showed that coating and charge influence toxicity through nonlinear interactions. Strongly cationic surfaces increased membrane affinity and promoted interparticle association and adverse cellular responses [8]. The model analysed 74 SPION formulations and predicted toxic potential for chitosan-based coatings with higher positive surface charge. It linked electrostatic surface state with biological interaction and aggregation propensity, supporting limits on strongly positive SPION surface charge. Deep learning analysis of transmission electron microscopy (TEM) images automated nanoparticle detection, segmentation, and size quantification [20]. The multiple-output convolutional neural network (MO-CNN) method achieved 98.23% detection accuracy and 96.59% segmentation accuracy on 17 TEM images of Fe_3_O_4_ and silica-coated nanoparticles.

Experimental studies clarified descriptor interpretation. Bare iron oxide nanoparticles disrupted lysosomal integrity and altered stress gene expression in cervical cancer cells [21]. Treatment with 100 μg/mL nanoparticles for 24 h significantly reduced cathepsin-B activity (*p* < 0.001). Transcriptomic analysis identified 179 differentially expressed genes, including 67 upregulated and 112 downregulated genes. These findings link lysosomal injury with broad transcriptional stress. Silica-coated magnetic nanoparticles produced lysosomal dysfunction and amyloid beta accumulation in microglia [22]. Accumulation increased significantly after exposure to 0.1 μg/μL rhodamine B isothiocyanate-labelled silica-coated magnetic nanoparticles (MNPs@SiO_2_(RITC)) compared with untreated cells and cells exposed to 0.01 μg/μL. The dose-dependent accumulation indicates that silica coating alone does not prevent adverse intracellular effects. AI/ML chromatin analysis detected hepatocyte nuclear alterations after iron oxide nanoparticle exposure that were not evident through conventional histopathology [23]. Random forest classification of 2000 hepatocyte nuclear regions achieved a test-set F1 score of 0.80 after mice received 2 mg/kg/day of 80–100 nm iron oxide nanoparticles for three days. Chromatin features can therefore reveal toxicity before conventional tissue changes.

Molecular dynamics (MD) simulations and differential scanning calorimetry (DSC) showed that polymer coatings impose distinct steric barriers and interfacial ordering effects [24]. Although both coated particles retained a hydrodynamic diameter of 12 nm, the polyvinyl alcohol (PVA)-coated magnetite core remained 8.6 ± 1.2 nm from the bilayer centre, compared with 10.6 ± 1.3 nm for the polyarabic acid (ARA)-coated core. The coatings included poly(polyethylene glycol methacrylate-*co*-acrylic acid) (P(PEGMA-AA)), PVA, and ARA. These effects altered particle–particle and particle–membrane interactions without changing the magnetic core. Nano-QSPR modelling showed that zeta potential is controlled by nonlinear coupling between environmental pH and nanostructure [4]. The consensus nano-QSPR model used 29 measurements from five metal oxides and achieved *R*^2^ = 0.912, with external predictive coefficients (QF1-3) > 0.897. Changes in pH affect electrostatic stabilisation and promote aggregation, making pH a key variable for predicting dispersion stability. Magnetic anisotropy and coercivity govern collective magnetic interactions among particles [25]. The exchange-bias model used 980 samples and 37 descriptors. XGBoost achieved *R*^2^ values of 0.74 and 0.75 on the test and independent validation sets, respectively. The moderate validation performance suggests that further descriptors are needed for collective magnetic behaviour.

Here, descriptors mean model-ready variables encoding nanocarrier structure, surface chemistry, colloidal state, magnetic behaviour, formulation conditions, biological exposure, or imaging response. Later sections distinguish stability, magnetic, process, biological, and image-derived descriptors by endpoint.

**3.1.2 Physicochemical mechanisms of colloidal stability and aggregation.** Surface charge and electrostatic repulsion depend on medium composition. Polymer coatings provide steric or electrosteric stabilisation. Near charge neutrality, reduced electrostatic repulsion increases aggregation risk. Ionic strength affects stability through charge screening and electric double-layer compression. pH and ionic strength should therefore be explicit model inputs rather than fixed experimental conditions [4]. The consensus nano-QSPR model predicted zeta potential from measured pH and structural descriptors for five metal oxides with *R*^2^ = 0.912. Its distilled-water dataset did not explicitly represent ionic strength or dissolved ions, limiting transferability to physiological ionic environments.

**Figure 2:**
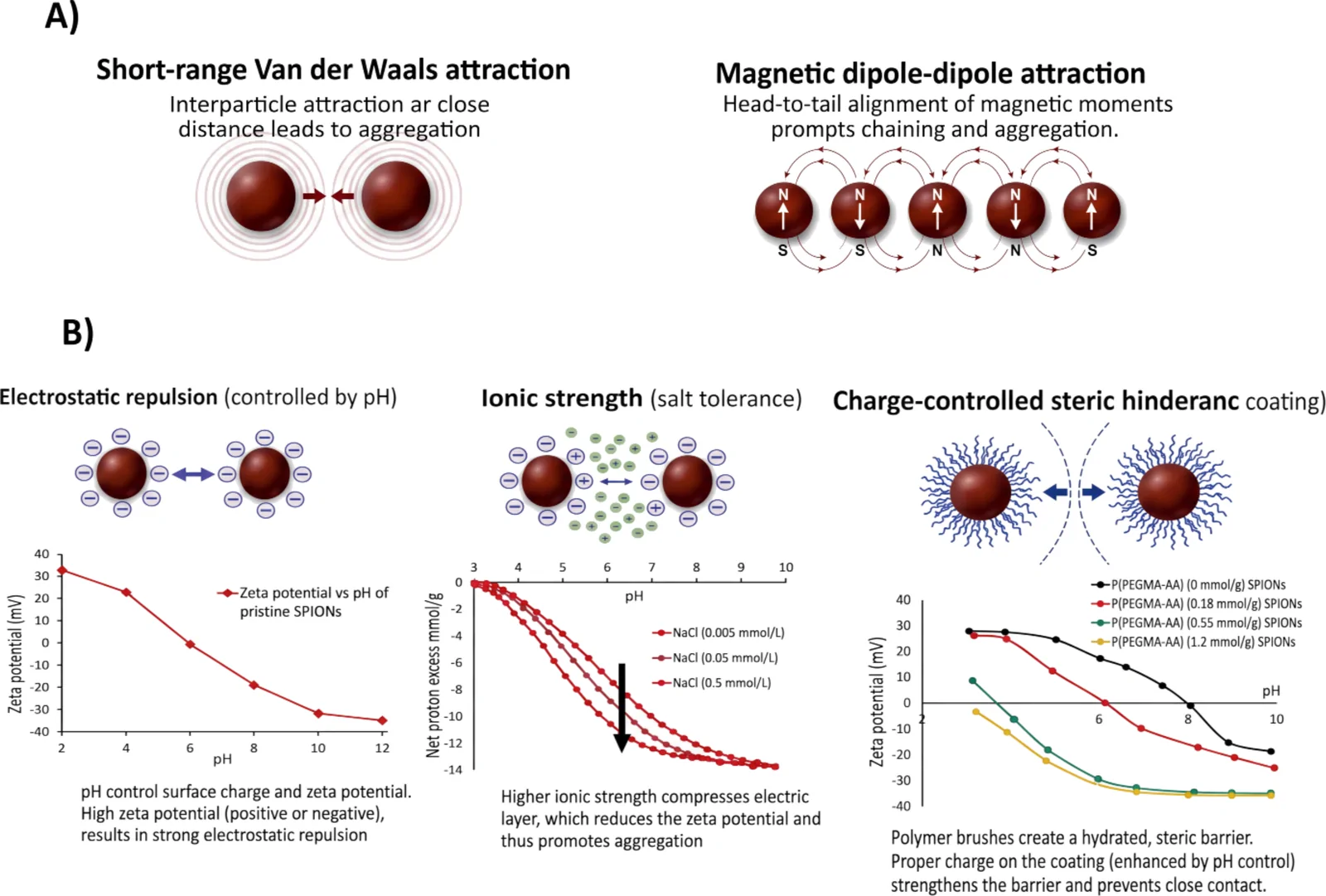
Destabilising interactions and stabilisation descriptors in magnetic nanoparticle colloidal stability. (A) van der Waals attraction and magnetic dipole–dipole coupling promote aggregation. (B) pH-dependent surface charging, ionic-strength charge screening, and P(PEGMA-AA)-mediated electrosteric stabilisation alter zeta potential and coagulation behaviour. The left graph in (B) was replotted from data reported in [27]. The middle and right graphs in (B) were replotted from data reported in [26].

Coating composition determines stabilisation around the magnetic core. In P(PEGMA-AA) coatings, carboxylate groups provide pH-dependent surface charge, whereas PEG chains provide a steric barrier. Polymer density and interfacial organisation remain important descriptors for electrosteric stabilization [24,26]. In membrane interaction simulations, a grafting density of three polymer chains per square nanometre defined complete coating of the 3 nm magnetite core and distinguished partial coverage from effective steric protection. Figure 2 links the main destabilising forces, van der Waals attraction and magnetic dipole–dipole coupling, with pH, ionic strength, zeta potential, and polymer stabilization [26–27]. This framework explains why aggregation cannot be represented by a single stability descriptor.

Exchange-bias modelling added magnetic descriptors to this framework. The ratio of Néel temperature (*T*_N_) to blocking temperature (*T*_B_) was identified as a predictor of interfacial magnetic coupling in heterostructured nanoparticles [25]. The model used the normalised temperature descriptor (*T*_N_ − *T*_B_)/*T*_N_ and was trained on 980 samples represented by 37 descriptors. This descriptor captures proximity to magnetic transitions independently of absolute temperature. Such interactions can strengthen chain formation or clustered states when electrostatic and steric barriers are insufficient. The descriptor hierarchy has three levels: Medium variables determine surface charging, coating variables determine steric and interfacial behaviour, and magnetic descriptors determine collective magnetic interactions.

**3.1.3 Process optimisation and coordinated stability-by-design.** Another area of investigation in this field is process optimisation. ANN models linked microfluidic flow rates and polymer concentrations with nanoparticle size distributions and relaxation behaviour in polymeric MRI systems [6]. The highest ANN accuracy was 98.9%. Antisolvent flow rate was the main size predictor in the less complex crosslinked hyaluronic acid nanoparticle system, identifying solvent mixing as a control point for particle-size regulation. The study mapped formulation and process variables to structural and imaging outputs.

In citrate-coated ferrofluids, a hybrid genetic algorithm-sequential quadratic programming (GA-SQP) approach optimised pH, citrate-to-Fe_3_O_4_ molar ratio, citrate volume, and coating temperature to maximise magneto-optical retardance [28]. The integrated GA-SQP method predicted a maximum retardance of 33.786° at pH 5.5, a citrate-to-Fe_3_O_4_ molar ratio of 0.12, a citrate volume of 40 mL, and a coating temperature of 70 °C. This defines a multivariable operating point for experimental verification.

Tree-based ensemble models in the DiMag platform integrated core and shell composition, particle geometry, coating features, magnetic parameters, nanoparticle concentration, and field conditions. The models predicted specific absorption rate (SAR) and MRI relaxivities [10]. Tenfold cross-validation produced *R*^2^ values of 0.86 for SAR, 0.78 for longitudinal relaxivity (*r*_1_), and 0.75 for transverse relaxivity (*r*_2_) using more than 980 magnetic nanoparticle records. The lower relaxivity scores indicate that MRI performance remains harder to predict than heat generation. This shared data structure linked stability-related formulation descriptors with magnetic performance outputs.

Hyperthermia models extended this approach to magnetic dissipation. AI/ML regressors included support vector regression, k-nearest neighbours, decision tree, random forest, gradient boosting, and ANN models. These models predicted SAR from core composition, particle diameter, saturation magnetisation, coercivity, nanoparticle concentration, and alternating-field amplitude and frequency [29]. ANN, gradient boosting, and random forest models outperformed support vector machine and k-nearest neighbour (KNN) models, with higher *R*^2^ values or lower prediction errors. A two-hidden-layer ANN performed comparably to deeper ANN models, indicating little benefit from added depth. A physics-informed neural network (PINN) predicted temperature-time profiles within a heat-balance constraint [30]. The PINN was trained on 3,690 data points and achieved a test-set *R*^2^ of approximately 0.98, compared with 0.96 for the ANN and 0.93 for random forest. Physical constraints improved prediction beyond purely data-driven modelling. The model represented magnetically induced heating, convective cooling, Brownian rotation, and Néel relaxation.

Most stability models were trained within bounded material and exposure spaces. The strongest direct evidence linked surface chemistry, particle size, hydrodynamic diameter, zeta potential, dose, and time with uptake or toxicity [14,31]. Stability-related performance therefore emerged from interacting formulation, environmental, and exposure variables. These findings support experimentally controllable descriptors in the stability-by-design space. Few models predicted aggregation or long-term colloidal stability as explicit endpoints. Stability was often inferred from downstream biological or magnetic performance rather than measured as a time-resolved outcome.

#### 3.2 AI and ML models for magnetic properties and MRI performance

**3.2.1 Prediction of magnetic descriptors.** Saturation magnetisation (*M*_s_), coercivity (*H*_c_), and remanent magnetisation (*M*_r_) link magnetic nanocarrier structure with imaging response. They describe magnetic magnitude and reversibility, and influence susceptibility effects, responsiveness, and field response. They do not determine MRI relaxivity alone.

A dataset of 1,758 characterised magnetic nanoparticles included Fe_3_O_4_, Fe_2_O_3_, and CoFe_2_O_4_ systems. Gradient boosting, XGBoost, and ExtraTrees models trained on this dataset predicted *H*_c_, *M*_r_, and *M*_s_ [9]. They achieved *R*^2^ values of approximately 0.78 for *H*_c_, 0.85 for *M*_r_, and 0.93 for *M*_s_. The higher *M*_s_ performance suggests that saturation magnetisation is more readily modelled than hysteresis-dependent properties.

A complementary ANN model using a multilayer perceptron (MLP), reported as ANN-MLP, predicted *M*_s_, *M*_r_, and *H*_c_ in Fe_3_O_4_@SiO_2_@MgAl layered double hydroxide (LDH) nanocomposites [32]. The two-hidden-layer MLP was trained on experimental data and 100 Monte Carlo-generated samples. It produced correlation coefficients above 0.98 for all outputs and low reported errors. Inputs were drying temperature, crystallite size, and particle size. Vibrating sample magnetometry data were augmented with spin-based Monte Carlo data.

Sensitivity analysis identified drying temperature as the dominant input [32]. Increasing drying temperature from 65 to 85 °C at pH 12 raised *M*_s_ from 24.01 to 28.14 emu/g and *H*_c_ from 57.27 to 72.46 Oe. These paired increases indicate stronger magnetisation and greater magnetic irreversibility. The result was consistent with effects on crystallinity, lattice strain, defect density, and magnetic dilution across the core–shell architecture. The study linked processing conditions and interfacial structure with magnetic outputs in a coated MRI-visible nanosystem whose core remained the main signal source.

Exchange bias prediction in heterostructured nanoparticles identified coercivity, anisotropy variables, and the Néel-to-blocking-temperature ratio as predictors of interfacial magnetic coupling [25]. XGBoost achieved *R*^2^ = 0.74 on the test set and *R*^2^ = 0.75 on an independent validation set of 51 samples. Coercivity, anisotropy field, and the normalised Néel-to-blocking-temperature descriptor were among the leading features. Validation supported prediction outside the training dataset, although variability in interfacial coupling remained incompletely explained.

**3.2.2 Prediction of relaxivity and MRI signal.** Hataminia and Azinfar modelled iron oxide contrast using SA-LOOCV-GRBF analysis [2]. The parallel SA-LOOCV-GRBF (PSLG) model evaluated nanoparticle sizes of 5, 11, and 31 nm and field strengths of 1.41, 4.2, and 11.7 T for prediction of 1/*T*_2_. Using SPION size, saturation magnetisation, nanoparticle concentration, and field strength as inputs, it expressed MRI output as 1/*T*_2_. The model captured nonlinear response surfaces across *M*_s_, size, concentration, and field strength, and tolerated synthetic noise better than random forest [2]. For noisy synthetic data, PSLG produced a mean squared error (MSE) of 0.457, compared with 16.06 for random forest. This supports PSLG noise tolerance, although testing used only synthetically generated noise. The model linked particle structure, intrinsic magnetism, dose, and acquisition field with MRI output.

The DiMag platform predicted *r*_1_ and *r*_2_ relaxivities [10]. The *r*_1_ and *r*_2_ models contained 355 and 465 samples, used eight descriptors, and achieved cross-validation *R*^2^ values of 0.78 and 0.75. These scores indicate that data harmonisation supported prediction but did not remove protocol variability. The database combined core and shell composition, particle geometry, coating descriptors, magnetic properties, concentration, and field conditions in a shared descriptor space for imaging and heating outcomes. Shapley additive explanations (SHAP) analysis identified whether composition, size, coating, magnetisation, concentration, or field variables dominated individual predictions, supporting interpretation at the level of adjustable design parameters.

DeepSPIO addressed SPION quantification rather than bulk relaxivity prediction. It used geometric distortions in View Line MRI as inputs to a residual encoder with multiple decoder branches [5]. Each decoder reconstructed a coefficient channel from the discrete wavelet decomposition of the SPION concentration map. The model reconstructed SPION concentration maps without directly fitting the transverse relaxation rate (*R*_2_) or solving phase-based quantitative susceptibility mapping. It targeted high SPION concentrations, where signal loss and phase unreliability limit conventional susceptibility-based methods. In validation experiments, DeepSPIO achieved an average normalised mean absolute error (NMAE) of 0.17 ± 0.10 on simulated data and estimated phantom SPION concentrations between 1.75 and 27.9 µg/mm^3^ with percentage errors of approximately 1.14–19.42% [5]. It linked image-domain distortions with tracer concentration and provided a downstream measure of SPION distribution. Reliability depends on distortions remaining within the training and validation domain.

Reinforcement learning was used to optimise SPION-based neurochemical MRI sensors [7]. The optimised 9 nm calcium sensor had a reaction rate constant of 0.156 s^−1^ and a response half-life of 4.44 s, compared with 0.066 s^−1^ and 10.5 s for the previous 12 nm sensor, indicating a more than twofold faster response. The workflow combined Monte Carlo MRI simulation with automated design search. SPION diameter, magnetisation, and three-dimensional sensor configuration were adjusted to maximise MRI contrast power and magnetic resonance signal change ratio. Experimental validation identified optimal diameters of 9 nm for calcium sensing and 11 nm for dopamine sensing, supporting transfer beyond simulation.

**Figure 3:**
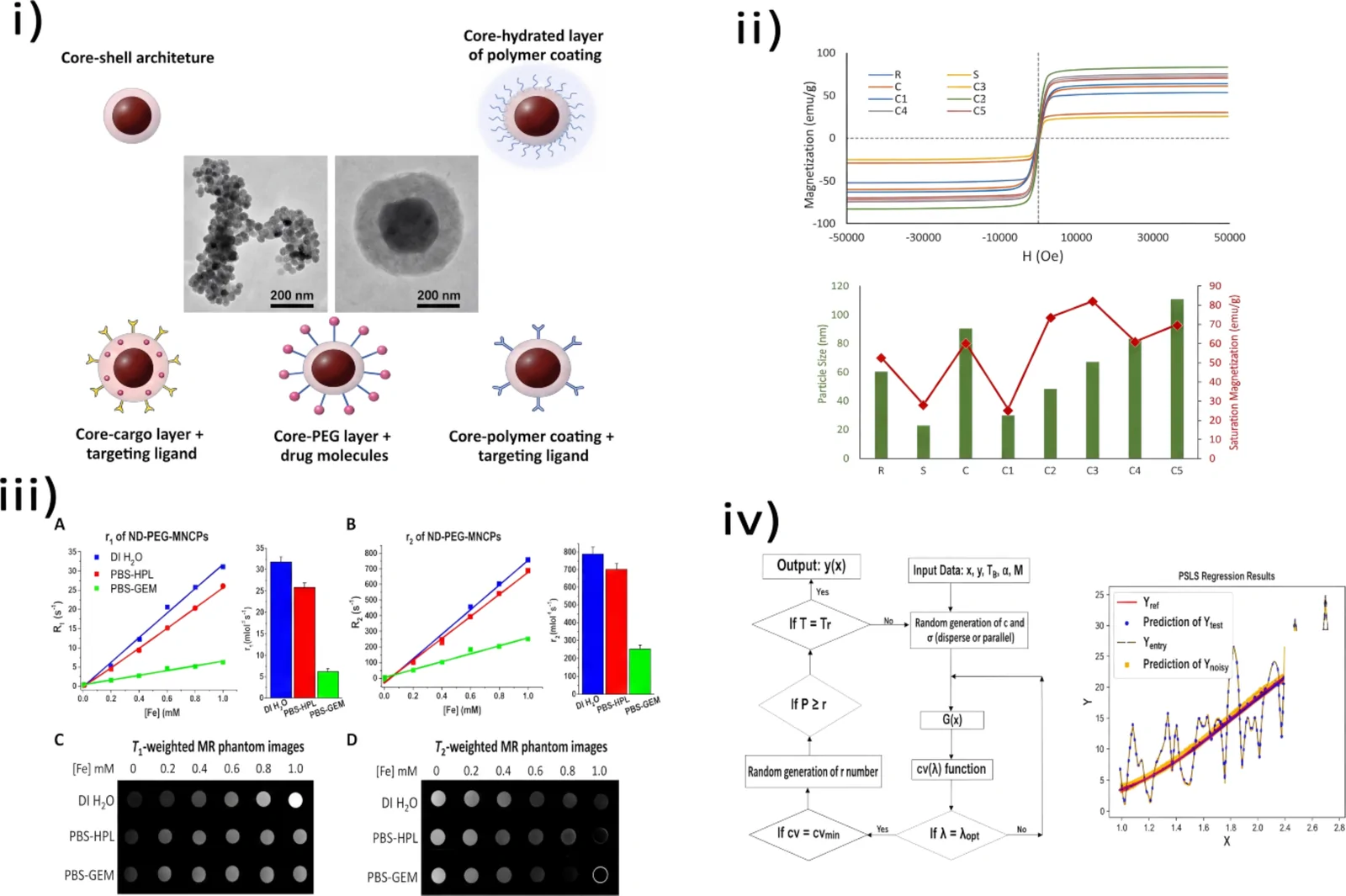
Structure–magnetism–MRI signal pathway for MRI-visible magnetic nanocarriers. (i) Representative magnetic-nanocarrier architectures; TEM images reproduced from [34], (“Quasi-Cubic Magnetite/Silica Core-Shell Nanoparticles as Enhanced MRI Contrast Agents for Cancer Imaging“, © 2011 J. L. Campbell et al., published by PLOS, distributed under the terms of the Creative Commons Attribution 1.0 Universal License, https://creativecommons.org/publicdomain/zero/1.0/). (ii) Magnetic-property data adapted from [1], © 2020 S. M. Dadfar et al., distributed under the terms of the Creative Commons Attribution 4.0 International License (https://creativecommons.org/licenses/by/4.0/). (iii) MRI relaxivity and phantom-imaging data adapted with permission of B. Thapa et al. from [33], (“T_1_- and T_2_‑weighted Magnetic Resonance Dual Contrast by Single Core Truncated Cubic Iron Oxide Nanoparticles with Abrupt Cellular Internalization and Immune Evasion”, B. Thapa, D. Diaz-Diestra, C. Santiago-Medina, N. Kumar, K. Tu, J. Beltran-Huarac, W. M. Jadwisienczak, B. R. Weiner, G. Morell, 1, © 2018); permission conveyed through Copyright Clearance Center, Inc. This content is not subject to CC BY 4.0. (iv) SA-LOOCV-GRBF modelling workflow and prediction output reproduced from [2] (© 2025 F. Hataminia et al., published by Springer Nature, distributed under the terms of the Creative Commons Attribution-NonCommercial-NoDerivatives 4.0 International License, http://creativecommons.org/licenses/by-nc-nd/4.0/). This content is not subject to CC BY 4.0.

Figure 3 summarises the structure–magnetism–MRI signal pathway including carrier architectures, size-dependent magnetic behaviour, *T*_1_- and *T*_2_-weighted outputs with relaxivity, and the SA-LOOCV-GRBF workflow [1–2,33–34].

**Figure 4:**
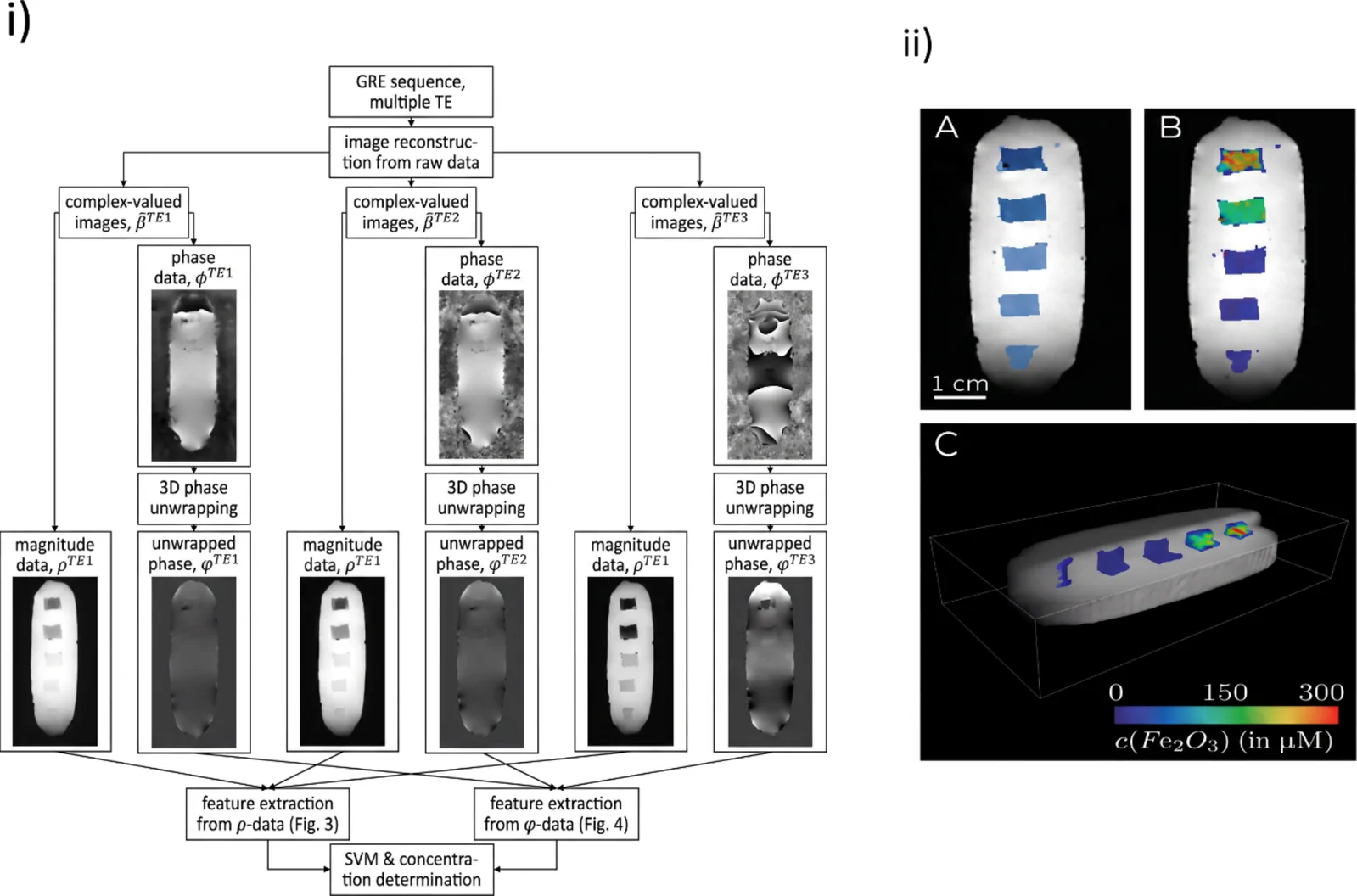
SVM-assisted MRI quantification of SPION-labelled cells. (i) Workflow for multiparametric MRI feature generation from multi-echo gradient-recalled echo (GRE) data. Complex-valued images from different echo times are reconstructed into magnitude and phase images, followed by phase unwrapping and extraction of *R*_2_* phase-based features for SVM-based concentration determination. (ii) Representative SVM output maps showing classified iron-positive regions and corresponding Fe_2_O_3_ concentration distributions in two-dimensional and three-dimensional views. Figure 4 was reproduced from [31]. (“In-Vivo Imaging of Cell Migration Using Contrast Enhanced MRI and SVM Based Post-Processing”, © 2015 C. Weis et al., published by PLOS, distributed under the terms of the Creative Commons Attribution 1.0 Universal License, https://creativecommons.org/publicdomain/zero/1.0/).

**3.2.3 AI-assisted quantification of SPION-labelled cells, tissues, and grafts.** Imaging studies used AI to detect, segment, or quantify SPION-labelled biological targets. In cell-tracking MRI, multi-echo gradient data were converted into voxel-wise magnitude, phase, and effective transverse relaxation rate (*R*_2_*) features for SVM analysis [31]. The SVM classified all iron-containing compartments in the training phantom and achieved a mean detected-to-actual volume ratio of 1.00 ± 0.03. It classified iron-positive voxels, generated labelled-cell maps, and estimated iron oxide concentration from *R*_2_*. The detection limit was 30 labelled cells per cubic millimetre, equivalent to 19 µM Fe_2_O_3_ [31]. It also identified 4 mg/mL Fe_3_O_4_ exposed to 15 kA/m at 320 kHz as suitable under the *H* × *f* safety limit of 5 × 10^9^ A·m^−1^·s^−1^. This workflow converted SPION-induced contrast into spatial estimates of labelled-cell or carrier distribution. Figure 4 illustrates the SVM quantification pipeline.

Afridi et al. used a two-step workflow for effective transverse relaxation time (*T*_2_*)-weighted MRI [35]. Superpixel extraction identified low-signal candidate regions, and transfer learning with a CNN classified them as magnetically labelled mesenchymal stem cells. The CNN achieved automated spot detection accuracies of 97.3% in vivo and 99.8% in vitro. The lower in vivo accuracy reflects more complex biological backgrounds. This workflow reduced manual spot identification and combined conventional image processing with deep learning for sparse labelled-cell signals.

Direct comparison within the same MRI dataset showed an advantage for the CNN over classifiers based on hand-crafted features. The CNN achieved the highest mean in vivo classification performance (94.6%), compared with 91.8% for random forest, 88.9% for multilayer perceptron, and 82.8% for naive Bayes [35]. On images acquired using a different MRI system and field strength, CNN and multilayer perceptron achieved accuracies of 97.3% and 95.3%, respectively [35]. These results support learned spatial features for sparse SPION detection, but do not establish general CNN superiority in magnetic imaging. SVM-based SPION quantification, DeepSPIO concentration mapping, and K-means++ MPI segmentation addressed different endpoints and validation settings [5,31,36], preventing direct numerical comparison across these tasks.

Molecularly targeted MRI extended this approach to tumour probe accumulation. In an orthotopic ovarian cancer model, under-glycosylated mucin 1-targeted magnetic nanoparticles generated negative *T*_2_ contrast [37]. Docetaxel-treated mice showed reduced magnetic nanoparticle-EPPT probe (MN-EPPT) accumulation relative to untreated controls, with *n* = 5 and *p* < 0.05. The reduction indicates sensitivity to treatment-induced changes in the targeted molecular phenotype. Deep learning segmentation defined tumour regions for change in *T*_2_ (Δ*T*_2_) analysis after chemotherapy. The AI component did not optimise nanoparticle formulation; it improved endpoint reproducibility for probe accumulation and treatment-associated change.

At higher iron concentrations, DeepSPIO reconstructed SPION concentration maps [5]. It achieved an NMAE of 0.17 ± 0.10 on simulated concentration maps similar to the training data. Nitrogen-vacancy-centre magnetic microscopy used simulated dipole fields and a CNN to estimate cell diameter, cell-to-sensor distance, and iron mass on the cell surface [36]. For a normalised 12 μm cell positioned 15 μm from the sensor, the predicted magnetic field reached 2.71 μT and approached a saturation value of 2.91 μT. The near-saturation response indicates diminishing sensitivity under this simulated geometry. This method is not conventional MRI, but it can validate cellular SPION loading and support MRI cell-tracking interpretation.

MPI studies provided a complementary measure of total iron in labelled grafts. K-means++ clustering segmented MPI signal from background in transplanted human islets [38]. A fiducial-based linear regression calibration curve converted segmented signal into total iron, since MPI volume alone cannot quantify retained mass. The same workflow was applied to three-dimensional MPI of SPION-labelled human stem-cell-derived islet organoids at 1, 7, and 28 days after transplantation [3]. MPI detected as few as 25 labelled islet organoids in vitro, and 800 organoids were used per in vivo transplant. This range extended the workflow to longitudinal graft monitoring. Phantom data showed a linear relation between iron content and organoid number. Automated segmentation was compared with manual radiologist segmentation using intraclass correlation coefficients (ICCs) [3]. K-means++ achieved ICCs of 0.898 for single measures and 0.927 for average measures. These values indicate good agreement and support reproducible automated MPI segmentation. These studies quantified SPION-labelled graft delivery and retention but did not optimise tracer design, so they were retained as downstream performance evidence.

Ferumoxytol-enhanced MRI provided a clinically oriented example of delivery-response mapping. In metastatic breast cancer, the spleen-normalised relative *R*_2_* 90th-percentile metric (r*R*_2_*,90), measured 16–24 h after ferumoxytol administration, predicted thoracoabdominal lesion response to liposomal irinotecan with 74% accuracy [39]. For brain metastases, the fraction of voxels with a ferumoxytol compartmentation index greater than 1 in the *R*_2_*,90 compartment (FCIGT1 *R*_2_*,90) achieved 79% cross-validation accuracy [39]. These moderate accuracies identify iron uptake as a candidate response biomarker requiring larger validation cohorts. The analysis used structured quantitative MRI metrics rather than an explicit magnetic nanocarrier design model. It showed that spatial and temporal iron oxide uptake can predict subsequent nanotherapeutic response.

#### 3.3 AI and ML models for integrated magnetic nanocarrier design and drug delivery

**3.3.1 Multivariable design frameworks.** Integrated design requires models that combine particle composition, coating, cargo, biological context, and magnetic or imaging performance.

The nanoparticle perturbation theory machine learning (NANO.PTML) framework combined information fusion, perturbation theory, and ML [40]. Its simulation space contained 2,607,600 combinations from five nanoparticle cores, 53 cell lines, five particle shapes, 16 coatings, and 123 drugs; most were not experimentally tested. Variables were core type, coating, shape, drug component, cell line, and assay conditions. Although developed for neuroscience rather than MRI-visible cancer nanocarriers, it is relevant because it jointly encoded formulation and biological variables.

A drug release ML framework compiled nanoparticle, drug, coating, and experimental descriptors [14]. Its dataset contained 714 nanoparticle drug delivery system instances. The optimised information fusion perturbation theory machine learning-linear discriminant analysis (IFPTML-LDA) model achieved sensitivity and specificity values of approximately 81.3–89.1% in the training and validation series. This suggests consistent classification across heterogeneous nanoparticle and drug combinations. The descriptors predicted release behaviour across diverse nanoparticle systems. The framework did not directly optimise MRI performance, but its multivariable structure is relevant to magnetic nanocarrier design.

These frameworks span a broader design space than the magnetic nanocarrier evidence reviewed here. Most magnetic studies optimised one dominant endpoint, that is, heating, size, release, transport, or targeting. To the authors’ knowledge, no supplied study jointly optimised stability, MRI contrast, toxicity, drug loading, and therapeutic response within one model. Multiobjective optimisation therefore remains the main unmet requirement.

**3.3.2 Magnetic core and synthesis optimization.** Magnetic hyperthermia contained the main data-driven examples. ANN-particle swarm optimisation models predicted and optimised temperature profiles from iron nanoparticle concentration, alternating magnetic field (AMF) conditions, and exposure time [41]. A concentration of 4 mg/mL exposed to 15 kA/m at 320 kHz produced heating within 42–47 °C while satisfying *H* × *f* < 5 × 10^9^ A·m^−1^·s^−1^. Thus, concentration and field parameters required joint optimisation, with the AMF product kept within a clinical safety limit.

A benchmarking study evaluated more than 20 ML regressors [42]. Three-layer and two-layer ANN models achieved adjusted *R*^2^ values of 0.9975 for steady-state temperature and 0.9561 for time to steady state, respectively, using 500 numerical simulations. The lower value indicates that transient heating is harder to predict. Field intensity, frequency, particle volume fraction, and particle diameter predicted steady-state tumour-centre temperature and time to steady state. Both studies integrated particle and field variables, but the outputs were thermal rather than MRI-based.

For α-Fe_2_O_3_ nanoparticles, grey relational analysis-adaptive particle swarm optimisation (GRA-APSO) and ANN modelling optimised synthesis variables [43]. Optimised and initial-condition particles had mean TEM sizes of 8.5 and 13.7 nm, respectively, while their *T*_2_ relaxivities were 34.8 and 36.4 mM^−1^·s^−1^. Size reduction did not improve *T*_2_ relaxivity, indicating misalignment between structural refinement and MRI performance. The models predicted crystallite size, lattice strain, crystallinity index, bond length, and bandgap. The particles were evaluated for haemocompatibility and MRI behaviour.

Support vector regression (SVR) predicted magnetic nanoparticle size from synthesis parameters [44]. Across eight regression methods, SVR produced the lowest root mean square error (RMSE) of 3.44, residual standard deviation of 5.13, and mean absolute percentage error (MAPE) of 0.25. The residual error indicates incomplete capture of synthesis-to-size relationships. The method was proposed for automated synthesis. In lithium-doped, chitosan-coated iron oxide nanoparticles, ML analysis identified material and synthesis variables associated with heating efficiency and SAR [45]. Li_1.5_Fe_1.5_O_3_ and chitosan-coated Li_1.5_Fe_1.5_O_3_ reached SAR values of 265.11 and 153.48 W/g, respectively, under a 278 kHz alternating field. Chitosan coating reduced SAR by approximately 42%, indicating a thermal cost of surface functionalisation. These studies used adjustable formulation or process variables to describe particle structure or magnetic performance.

Nonlinear autoregressive with exogenous inputs-long short-term memory (NARX-LSTM) models predicted temperature evolution in graphene–magnetite nanohybrids across composition and AMF conditions [46]. Adaptive neuro-fuzzy inference system (ANFIS) models predicted the heating response of pH-responsive graphene oxide–iron oxide hybrids as a function of time, composition, and pH [47]. The subtractive clustering ANFIS model achieved a testing *R*^2^ of 0.9938 and an MSE of 0.03097 across pH values of approximately 4, 5, 7, and 10. pH-dependent heating was therefore predictable within the sampled conditions.

Earlier nonlinear autoregressive with exogenous inputs (NARX) modelling described heat induction in graphene-Fe_3_O_4_ nanohybrids [48]. The F45G55 nanohybrid produced the highest SAR of 6.45 W/g, compared with 4.32 W/g for pure magnetite, and its NARX model reached an MSE of 0.44703 after 71 epochs. The approximately 49% SAR increase indicates a heating benefit from graphene incorporation. These models incorporated environmental and compositional inputs, but the outcome remained magnetic heating. Their relevance to MRI-visible drug delivery depends on using the same hybrid architecture for magnetic actuation, imaging, and therapy.

**3.3.3 Polymeric, fibrous, and gel-based magnetic reservoirs.** Polymeric magnetic reservoirs provided direct drug delivery evidence. In poly(*N*-isopropylacrylamide-*co*-vinylsulfonic acid)-Fe_3_O_4_ interpenetrating polymer network ferrogels, ANN models predicted deswelling and temperature rise at different AMF strengths [49]. The ferrogels contained Fe_3_O_4_ nanoparticles with an average size of 13 nm and were tested at 1.37, 1.64, and 1.91 mT. These values define the modelled operating range, not predictive accuracy. The model linked composition and magnetic stimulation with dimensional response and local heating, both relevant to reservoir function.

A reduced graphene oxide (rGO)–Fe_3_O_4_ ferrogel study used ANN modelling to predict doxorubicin (DOX) loading and release under alternating magnetic fields [50]. At 1.64 mT, equilibrium DOX release across six formulations ranged from 79% to 85%, suggesting limited formulation influence at a fixed field strength. Inputs were rGO content, magnetic heating, and AMF strength. The model linked carrier composition and external stimulation with release behaviour, combining magnetic material, cargo, stimulus, and release endpoint.

In biodegradable PVA-nanohydroxyapatite-Fe_3_O_4_ scaffolds, ANN models predicted swelling, degradation, porosity, and mechanical behaviour from composition and environmental conditions [51]. The optimised ANN achieved a cross-validation *R*^2^ of 0.96 and an average absolute error below 5%. The highest loaded scaffold reached 176% swelling, 72.7% porosity, 4.61 MPa compressive strength, and 2.13 GPa elastic modulus. Predicting swelling, porosity, and mechanics supports assessment of formulation trade-offs before implantation. Drug release was not the principal output; the predicted properties described transport, structural integrity, and implantation performance.

In 5-fluorouracil (5FU)-loaded polycaprolactone (PCL)–chitosan–Fe_3_O_4_ core–shell nanofibres, ANN modelling estimated release parameters [52]. The ANN predicted release parameters with approximately 10% error. A genetic algorithm selected an optimum formulation containing 22% chitosan, 5.9% Fe_3_O_4_, and 4.6% 5FU. The remaining error means that the formulation still requires experimental confirmation. The workflow combined magnetic carrier composition, drug cargo, release kinetics, and formulation optimisation.

Predictive performance in drug-delivery modelling was also model- and endpoint-dependent. In the 714-system IFPTML dataset, 11 classifiers were evaluated under three-fold cross-validation [14]. Random forest achieved the highest mean AUROC (0.9973), closely followed by XGBoost (0.9970), whereas LDA achieved 0.9912. Despite the marginally higher ensemble performance, the simpler IFPTML-LDA framework was retained because it provided comparable discrimination with lower model complexity; external validation was not available. In magnetic delivery systems, ANN modelling of biodegradable scaffolds achieved a cross-validation *R*^2^ of 0.96 and an average absolute error below 5% [51], while ANN-guided optimisation of 5FU-loaded magnetic nanofibres produced release-parameter errors of approximately 10% [52]. These results cannot be ranked directly because the studies differed in endpoints, datasets, and validation schemes. Thus, ensemble methods performed strongly in the broader drug-delivery classification task, whereas the available magnetic-carrier studies support ANN-based local optimisation without establishing algorithm-level superiority.

**3.3.4 Surface chemistry, targeting, and nano-bio interfaces.** Surface engineering affects dispersion, nonspecific interactions, target binding, and cargo release. In receptor-deficient triple-negative breast cancer, covalent organic framework (COF)@DOX@Fe_3_O_4_ nanocarriers increased uptake under magnetic guidance and achieved DOX loading of 24.53 ± 0.09 wt % [53]. Magnetic targeting reduced the half-maximal inhibitory concentration (IC_50_) to 0.189 μM in MDA-MB-453 cells and 0.0341 μM in 4T1 cells. The approximately 5.5-fold lower IC_50_ in 4T1 cells indicates a cell-line-dependent therapeutic response. The system combined COF-based loading, Fe_3_O_4_-mediated targeting, pH-responsive release, and reduced off-target toxicity.

A C-X-C chemokine receptor type 4 (CXCR4)-targeted core–shell study used computational prediction of drug synergy, peptide ligand discovery, and formulation design [54]. Its dual-AI screen identified a nine-residue CXCR4-binding peptide with an interprotein template modelling score (TM-score) of 0.96 and predicted aligned error below 10 Å at the receptor interface. These metrics support structural plausibility, not experimental peptide binding efficacy. Computational targeting remains incompletely linked to magnetic nanocarrier implementation. Magnetic systems often demonstrate targeting, uptake, or release experimentally; AI-guided ligand discovery and multidrug optimisation are reported more often for non-magnetic carriers. Integrated magnetic core design, MRI contrast, ligand density, drug synergy, and tumour-specific uptake remains uncommon, preventing optimisation of targeting against imaging and colloidal stability trade-offs.

Table 1 summarises design patterns across magnetic core optimisation, hybrid magnetic reservoirs, drug release systems, synthesis optimisation, and nano-bio interface engineering, and classifies endpoint integration.

**Table 1:** AI/ML-driven co-design patterns in magnetic nanocarrier optimisation. The table summarises recurring design strategies by mapping core, coating, and cargo variables to delivery and magnetic and imaging endpoints, together with coupling level, optimisation type, and applied AI frameworks, highlighting the limited integration of delivery and imaging within unified optimisation approaches.

Design pattern	Core, coating, and cargo	Delivery endpoints	Endpoints	Coupling type	Optimisation type	AI frameworks	Key limitation	Ref.

magnetic core optimisation (hyperthermia-focused)	core: Fe_3_O_4_ size, composition, doping; coating: minimal; cargo: none	not modelled	SAR, temperature rise	independent	single-objective	ANN, ANN-PSO, NARX, CART	no delivery integration	[39–40,44,52]
core biocompatibility and relaxivity tuning	core: crystallinity; coating: none; cargo: none	haemocompatibility	*T*_2_ relaxivity	weakly coupled	implicit multitarget	GRA-APSO-ANN	no joint optimisation	[43]
hybrid magnetic-polymer systems (ferrogels)	core: Fe_3_O_4_; coating: polymer/rGO; cargo: DOX (partial)	swelling, drug release	temperature rise	weakly coupled	implicit multitarget	ANN	no explicit multiobjective optimisation	[49–50]
polymeric scaffold drug carriers	core: Fe_3_O_4_; coating: PVA, PCL and CS; cargo: 5FU	release kinetics	not modelled	independent	single-objective	ANN	no magnetic and imaging coupling	[51–52]
synthesis and process parameter optimization	core: synthesis conditions; coating: none; cargo: none	reaction yield (proxy)	not modelled	independent	single-objective	evolutionary ANN, SVR	no biomedical endpoints	[44,55]
ligand, targeting, and nano-bio interface modelling	core: Fe_3_O_4_; coating: ligands (HA, CXCR4, COF); cargo: drugs	uptake, targeting, drug synergy	magnetic guidance	weakly coupled	implicit multitarget	AI-guided design, docking	no imaging optimisation	[53,56]
bio-response and immunological profiling	core: Fe_3_O_4_; coating: surface-modified, Au, and MXene; cargo: none	immune response, classification outputs	not modelled	independent	implicit multitarget	ML clustering, PCA-RF, FCNN	no design-variable optimisation	[56–59]

**3.3.5 Pharmacokinetics, transport, and tumour localization.** Pharmacokinetic (PK) and transport models connect formulation variables with in vivo delivery. A three-compartment PK model was applied to erythrocyte membrane-camouflaged magnetofluorescent nanocarriers [60]. The nanocarrier showed a 92 h elimination half-life and approximately 10% injected-dose tumour delivery efficiency in the Ehrlich murine tumour model. The prolonged half-life supported sustained exposure, but most of the injected dose remained outside the tumour. Input data came from fluorescence molecular tomography and alternating-current biosusceptometry. The model represented blood clearance, nonspecific tissue accumulation, tumour uptake, and estimated delivery efficiency and time to maximum tumour concentration. The study quantified magnetic carrier distribution rather than treating biodistribution as descriptive.

ML surrogate models approximated magnetic nanoparticle transport in blood-flow simulations by predicting trajectories or deposition under magnetic-field conditions. A Levenberg–Marquardt neural network (LM-NN) predicted nanoparticle concentration and transport behaviour under magnetic fields [61]. The feedforward LM-NN contained two input neurons, ten hidden-layer neurons, and one output neuron, and was trained on simulation-derived data from a coupled cardiovascular transport model. Regression coefficients were 1.00, 0.99993, 0.99999, and 0.99993 for training, validation, testing, and overall performance, respectively. Another ANN study modelled nanoparticle-mediated drug delivery in pulsatile flow [62]. The LM-NN used one hidden layer with ten neurons and a 70:30 training-to-validation split for simulation-derived velocity profiles. This split evaluates numerical interpolation rather than transfer to experimental haemodynamics. Hybrid ML was also used to analyse ferrohydrodynamic blood flow containing magnetic nanocarriers [63]. The k-nearest neighbour, gradient boosting, and decision tree models achieved *R*^2^ values of 0.99088, 0.96168, and 0.90278, respectively, for blood velocity prediction. The higher KNN performance suggests that local interpolation captured the simulated velocity field. These studies enabled exploration of field, flow, particle, and concentration variables, but their validity depends on the geometry, boundary conditions, rheology, and particle-fluid assumptions used to generate training data.

Rule-based image analysis connected tumour geometry with particle-size recommendation. You Only Look Once version 8 (YOLOv8) detected liver tumours in MRI and extracted size features [64]. The detector achieved 99.7% precision, 98.6% recall, mean average precision at an intersection-over-union threshold of 50% (mAP@50) of 99.5%, and mean average precision across 50–95% thresholds (mAP@50-95) of 91.9%. The lower mAP@50-95 indicates reduced localisation precision under stricter overlap thresholds. Predefined rules then generated nanoparticle size recommendations. The workflow shows how patient imaging features can be incorporated into a design decision pipeline.

**3.3.6 Magnetic navigation and externally controlled delivery.** Data-driven control models treated magnetic-field inputs and particle trajectories as coupled input–output relationships, allowing particle motion to be learned, predicted, and optimised from training data. Van Durme et al. trained neural networks to predict magnetic particle cluster velocity from cluster position and electromagnet currents in an in vitro targeting system [65]. The best network used two 32-neuron rectified linear unit (ReLU) hidden layers and achieved *R*^2^ values of approximately 0.92 for training data and 0.90 for test data. The small performance gap suggests limited overfitting in this controlled in vitro setting. The surrogate model was embedded in trajectory optimisation, where it guided particle motion while reducing energy dissipation. By approximating the relationship between magnetic-field inputs and particle motion, the surrogate reduced the need for repeated solution of the full transport model during optimisation.

ANN inverse design determined the rotating magnetic field parameters needed to generate predefined elliptical magnetic nanoparticle swarm geometries [66]. The model was trained on 144 rotating-field combinations and produced RMSE values of 0.21 mm, 0.06 mm, and 6.01° for the swarm major axis, minor axis, and slant angle, respectively. It linked desired swarm shape and orientation with field-control variables, enabling automated configuration of collective particle behaviour.

Water-stable magnetic lipiodol microdroplets combined SPION-containing droplets with AI-directed closed-loop navigation [67]. The SPIONs had an approximate diameter of 150 nm, and the microdroplets retained stable morphology in saline and blood for more than seven days. This morphology supports short-term navigation, but morphology alone does not establish colloidal integrity. Visual feedback supported path recognition, obstacle avoidance, and magnetic motion planning. The system combined a magnetic drug-delivery vehicle with image-based control and real-time navigation.

AI-based computer vision quantified intracellular doxorubicin release and retention from cancer-cell-membrane-camouflaged Fe_3_O_4_ nanobot assemblies [68]. At 48 h, DOX fluorescence intensity in cells treated with membrane-camouflaged nanobots was approximately 2.9-fold higher than after free DOX treatment. Sustained fluorescence indicates enhanced intracellular retention but does not independently quantify released DOX. The model assessed therapeutic delivery after actuation rather than controlling particle motion.

Algorithm comparison was stronger for magnetic transport prediction than for navigation control itself. In ferrohydrodynamic blood-flow modelling, KNN achieved *R*^2^ = 0.99088, exceeding gradient boosting (*R*^2^ = 0.96168) and decision-tree regression (*R*^2^ = 0.90278) on the same simulated dataset [63]. In contrast, navigation studies generally evaluated a single modelling framework. The neural-network trajectory model achieved a test *R*^2^ of approximately 0.90 [65], while ANN-based swarm inverse design produced RMSE values of 0.21 mm, 0.06 mm, and 6.01° for the major axis, minor axis, and orientation, respectively [66]. These results demonstrate high task-specific predictive performance but do not establish algorithm superiority for magnetic navigation because the models addressed different control outputs and were not benchmarked on a common navigation dataset. Direct head-to-head evaluation under matched experimental conditions remains lacking.

These studies show that AI contributes to magnetic-field-guided delivery of therapeutic magnetic nanocarriers to tumour regions through transport prediction under flow and field conditions, inverse design of control parameters, and image-based localisation or release assessment. Their shared limitation is limited integration with MRI relaxivity and colloidal stability.

#### 3.4 Data structure, model inputs, and evidence quality

**3.4.1 Dataset origins and scale.** Model performance was shaped strongly by data provenance. Controlled laboratory datasets usually produced the clearest structure–property relationships, but often remained confined to one formulation space. Larger curated datasets broadened chemical and experimental coverage. Simulated and image-derived datasets sampled or quantified variables difficult to measure experimentally. Each stream therefore enabled different modelling and validation claims.

Single-laboratory datasets were used for microfluidic formulation, polymer matrix MRI modelling, ferrogel release, scaffold mechanics, synthesis optimisation, and magnetic heating [28,36,40,43,46]. These datasets used consistent materials and protocols but restricted design spaces. High performance within narrow ranges of polymer concentration, crosslinker content, particle size, pH, or field conditions does not establish transferability. These models are better interpreted as local optimisers than transferable predictors.

DiMag aggregated particle composition, geometry, coating, magnetic, field, SAR, and relaxivity data across studies [10]. Its initial database contained 1,282 samples from 126 scientific articles before cleaning and endpoint-specific dataset construction. A separate multimaterial magnetic-descriptor study compiled 1,758 nanoparticle records to predict *H*_c_, *M*_r_, and *M*_s_ [9]. Because records differed by endpoint, the study used 1,298 samples for *H*_c_ and *M*_r_ modelling but only 460 samples for *M*_s_ modelling. This imbalance means that performance differences partly reflect data availability rather than algorithm quality alone. Cross-study toxicity models combined particle descriptors with exposure context, assay conditions, and biological endpoints to predict safety across heterogeneous settings [18–19].

Publicly available data resources differ substantially in scope. DiMag is the most directly relevant magnetic-specific resource identified, providing curated datasets and model code for SAR and MRI relaxivity prediction. By comparison, eNanoMapper is a broader public nanomaterial database containing physicochemical characterisation and biological and toxicological data, making it more applicable to nanosafety modelling than to magnetic or MRI-specific benchmarking [69]. PubVINAS similarly provides public nanoinformatics data spanning multiple nanomaterial types and physicochemical and biological descriptors, but is not designed around magnetic-nanomedicine endpoints [70]. Thus, no current public benchmark integrates magnetic properties, relaxivity, colloidal stability, drug delivery, and safety using standardised metadata and common validation partitions. DiMag provides the closest domain-specific resource, while broader nanoinformatics databases provide complementary biological and safety information.

Several hyperthermia and synthesis datasets combined different particle sizes, coatings, concentrations, field conditions, and reporting conventions [29,44]. Chu et al. compared support vector, nearest neighbour, ensemble, and neural network regressors using particle diameter, *M*_s_, *H*_c_, concentration, field amplitude, and frequency as predictors of heating performance [29]. Glänzer et al. found that support vector regression produced an RMSE of 3.44, a residual standard deviation of 5.13, and a mean absolute percentage error of 0.25 for nanoparticle size prediction [44]. These datasets increased chemical and structural diversity, but missing descriptors and protocol inconsistency can produce predictive patterns that partly reflect study origin.

Simulated data were used when experimental sampling was expensive or systematic field, geometry, or flow variation was required. In the Fe_3_O_4_@SiO_2_@MgAl-LDH study, spin-based Monte Carlo simulation added 100 generated cases to the experimental dataset used for ANN-MLP prediction of *M*_s_, *M*_r_, and *H*_c_ [32]. This augmentation increased the number of training examples, but added cases reflected the simulation’s magnetic assumptions. Monte Carlo MRI simulation was also used in reinforcement-learning optimisation of SPION-based neurochemical sensors [7]. The predicted designs were experimentally tested at selected particle diameters, identifying 9 nm SPIONs for calcium sensing and 11 nm SPIONs for dopamine sensing. This confirmed selected optima experimentally, although it did not validate the full simulated design space. Transport and control studies used a related strategy: Neural-network models were trained on numerical flow fields, computational fluid dynamics (CFD)-derived velocity data, tumour-image features, or controlled magnetic navigation experiments [62–63,65–66]. These datasets explored pulsatile flow, ferrohydrodynamic transport, tumour-size design rules, particle–cluster navigation, and rotating-field swarm control. Their predictive value remains tied to assumptions about vessel geometry, boundary conditions, fluid rheology, magnetic-field homogeneity, particle interactions, image quality, and actuation hardware.

Imaging datasets supported SPION localisation and concentration mapping. SVM-based cell tracking used magnitude, phase, and *R*_2_* features [31]. The method detected 30 labelled cells per cubic millimetre, corresponding to approximately 19 μM iron oxide, in phantom experiments. CNN models analysed candidate spots in *T*_2_*-weighted MRI, while DeepSPIO used image distortions [5]. On simulated concentration maps resembling its training distribution, DeepSPIO achieved an NMAE of 0.17 ± 0.10. This performance positions DeepSPIO as a quantitative accumulation tool rather than a detection-only method. Targeted MRI used segmented tumour regions and Δ*T*_2_ measurements [37]. This study analysed five animals per group and detected reduced targeted probe accumulation after docetaxel treatment at *p* < 0.05. These studies show that AI can convert SPION-induced contrast into quantitative biological readouts, but generalisability depends on validation across broader subject numbers, scanners, pulse sequences, and signal conditions.

**3.4.2 Descriptor engineering and model-ready variables.** Descriptor engineering determined how each study translated nanoparticle design, experimental context, and imaging response into model-ready variables. The reviewed models did not share a single descriptor framework. Descriptor choice followed the endpoint being predicted, including magnetic behaviour, relaxivity, toxicity, uptake, release, heating, transport, or image-derived SPION burden. This section examines which physical, chemical, biological, and acquisition variables were made visible to the models and where descriptor construction remained insufficiently reported.

Descriptor construction determined which mechanisms each model could learn. In magnetic-property and MRI-performance studies, descriptors represented core architecture and field response, that is, elemental ratios, core type, shell composition, particle diameter, crystallite size, geometry, *M*_s_, *M*_r_, *H*_c_, magnetic moment, exchange-bias field, anisotropy-related variables, concentration, and field strength [6,15]. These variables connected composition and structure with hysteresis, SAR, relaxivity, and MRI signal, but did not by themselves describe colloidal state or biological exposure [2,9–10,25,29,32].

Biological-response and toxicity models used a different descriptor logic. Hydrodynamic diameter, PDI, zeta potential, coating chemistry, molecular properties of surface modifiers, pH, dose, incubation time, cell type, toxicity endpoint, assay conditions, and PEG-coating status represented the particle state encountered by cells [14,16,31,50]. The same magnetic core may therefore produce different biological responses after changes in coating, aggregation state, charge, dose, or exposure duration.

Process-optimisation and drug-delivery models used descriptors directly adjustable during synthesis or formulation. These included polymer and crosslinker concentration, microfluidic flow rates, drying temperature, citrate ratio and volume, coating temperature, magnetic-particle content, drug loading, and hybrid-material composition [40,43,45–47]. Such variables supported optimisation because they linked model outputs to experimental control points rather than only to final particle measurements.

Image-derived descriptors served a separate function. *R*_2_*, Δ*T*_2_, compartmentation metrics, and calibrated total iron values converted MRI and MPI signal changes into estimates of SPION burden, probe accumulation, graft retention, or delivery response [3,31,37–39]. These features made biological localisation measurable, but reliability depended on segmentation, calibration, scanner conditions, and acquisition protocols.

Exposure and acquisition variables were useful when they connected material properties to measurement conditions. Dose and incubation time made toxicity models sensitive to biological exposure. Magnetic-field strength, AMF parameters, concentration, and pulse-sequence-derived measurements allowed heating and imaging models to account for externally imposed conditions [2,5,7,10,29–31,35,37].

Descriptor selection and interpretation differed substantially across modelling approaches. In zeta-potential nano-QSPR modelling, GA-PLS selected latent-variable combinations from physicochemical descriptors; pH was retained across the individual models, while DFT-derived descriptors contributed strongly to the fitted relationships [4]. In SPION coating-toxicity modelling, PCA ranked descriptors through component loadings, whereas PLS used variable-importance-in-projection scores to identify influential predictors, including descriptors related to hydrophobicity, molecular connectivity, and charge polarisation [8]. Tree-based magnetic-property models instead used engineered physicochemical descriptors followed by SHAP analysis: Exchange-bias field dominated *H*_c_, *M*_s_, and *H*_c_ together with elemental ratios influenced *M*_r_, and core magnetic moment and geometric anisotropy governed *M*_s_ [9]. DiMag similarly combined curated and engineered composition and structural descriptors with SHAP interpretation for SAR and relaxivity prediction [10]. By contrast, the ANN-MLP model used a small predefined input set and sensitivity analysis, identifying drying temperature as more influential than crystallite size and particle size [32]. These approaches serve different purposes: PCA and PLS reduce or select correlated descriptor spaces, SHAP attributes importance within fitted nonlinear models, and sensitivity analysis evaluates the response to predefined inputs. Feature rankings are therefore endpoint- and model-dependent and should not be interpreted as universal mechanistic hierarchies.

**3.4.3 Validation and model performance.** Validation practices varied substantially. Stronger tabular studies separated training and evaluation data, compared algorithms, used cross-validation, and reported multiple performance metrics.

Chen et al. reported *R*^2^ values for *H*_c_, *M*_r_, and *M*_s_ [9]. Gradient boosting achieved test-set *R*^2^ values of 0.78 for *H*_c_ and 0.85 for *M*_r_, while ExtraTrees achieved *R*^2^ = 0.93 for *M*_s_ after an 80:20 split and fivefold cross-validation. This design reduces split-specific optimism, although all evaluation records came from the same curated dataset. The study also used SHAP analysis for model interpretation. Shirmahd et al. reported correlation coefficients above 0.98 and included sensitivity analysis [32]. The model achieved its best validation MSE of 0.04216 at epoch 83, with training, validation, test, and overall correlation coefficients of 0.99172, 0.98982, 0.98641, and 0.98861, respectively. The small decline from training to test performance suggests limited overfitting within the combined dataset. DiMag used ensemble models and SHAP interpretation across SAR and relaxivity outputs [10]. Tenfold cross-validation produced *R*^2^ values of 0.86 for SAR, 0.78 for *r*_1_, and 0.75 for *r*_2_, indicating moderate to strong internal predictive performance. Because the database was assembled from literature sources, similar formulations or related experimental records may have been distributed across folds. This could make cross-validation more optimistic than validation on a fully independent external dataset.

The MRI signal model tested noise tolerance by comparing SA-LOOCV-GRBF with random forest under synthetic noise [2]. PSLG produced an MSE of 0.457, compared with 16.06 for random forest, giving an approximately 35-fold error reduction under the imposed noise model. Validation was more clinically anchored in imaging studies, where models were assessed using held-out data, cross-validation, phantom calibration, or expert segmentation. Weis et al. achieved a detection limit of 30 labelled cells per cubic millimetre, corresponding to 19 μM iron oxide, using an SVM trained on multiparametric phantom data [31]. This threshold supports method calibration but should not be generalised across scanners or pulse sequences. Afridi et al. achieved automated spot detection accuracies of 97.3% in vivo and 99.8% in vitro with a deep CNN [35]. The close in vitro and in vivo performance indicates consistency across these two imaging settings. Zhao et al. compared AI-generated tumour regions with board-certified radiologist segmentations using ICC analysis and evaluated five animals per treatment group [37]. Radiologist comparison provides reference-based validation, although five animals represent limited biological heterogeneity. External multicentre validation remained uncommon.

In ferumoxytol-enhanced MRI, cross-validation produced 74% accuracy for thoracoabdominal lesions and 79% for brain metastases [39]. The analysis included 49 thoracoabdominal metastases from 19 participants and 19 brain metastases from seven participants. Unless validation was participant-stratified, multiple lesions per patient could inflate apparent independence. MPI graft studies compared automated and manual segmentation using ICCs [3]. K-means++ segmentation achieved ICCs of 0.898 for single measures and 0.927 for average measures. The higher average-measures coefficient indicates that aggregation improved segmentation reproducibility.

To permit method-level comparison without conflating results from non-equivalent datasets, Table 2 summarises only within-study benchmarks in which competing algorithms were evaluated for the same endpoint using the same dataset.

**Table 2:** Within-study comparison of artificial intelligence and machine-learning algorithms across representative magnetic nanoparticle prediction tasks. Performance values are reported only for algorithms evaluated on the same dataset within the same study to avoid invalid cross-study ranking. Dataset size, validation approach, performance metrics, the best-performing method, and the principal limitation of each comparison are summarised.

Endpoint	Algorithms compared	Dataset size	Validation method	Performance metric	Best-performing method	Key limitation	Ref.

SAR and magnetic hyperthermia	SVR, KNN, decision tree, random forest, gradient boosting, ANN	626 records from 56 papers; 44 MNP compositions	80:20 train and test split; fivefold CV and grid-search tuning; repeated random states	*R*^2^, MAE, RMSE	ANN showed the highest predictive performance, followed by random forest and gradient boosting; two hidden ANN layers were sufficient	literature-derived data were heterogeneous, imbalanced, and contained outliers; no independent external experimental dataset	[29]
temperature-time profile during magnetic hyperthermia	linear regression, SVR, KNN, polynomial regression, gradient boosting, decision tree, random forest, ANN, PINN	3,690 records from eight in-house studies	held-out train and test evaluation; physical parameters calibrated using training data only	*R*^2^, RMSE	PINN: *R*^2^ = 0.98, RMSE = 0.61; ANN: 0.96, 1.10; random forest: 0.93, 1.42	data originated from the same research programme and instrumentation; digitisation noise and a restricted field-frequency range were reported	[30]
MNP size from synthesis conditions	ridge, lasso, elastic net, decision tree, random forest, gradient boosting, SVR, multilayer perceptron	113 initial records; 67 retained after filtering and outlier removal	LOOCV for hyperparameter optimisation followed by a random 80:20 final split	RMSE, MAE, MAPE, residual SD	SVR: RMSE = 3.44 nm, MAPE = 0.25, residual SD = 5.13	small, highly filtered, synthesis-setup-specific dataset; transferability to other synthesis systems was not established	[44]
MRI transverse relaxation rate (1/*T*_2_)/noise robustness	PSLG and random forest; DSLG also assessed during model development	117 MRI records from 15 articles (89 training, 28 testing); separate *n* = 50 synthetic benchmark for direct PSLG-RF comparison	train and test evaluation of MRI model; separate clean and noise-added synthetic benchmark	MSE	condition-dependent: RF performed better on clean synthetic data (0.017 vs 0.236), whereas PSLG performed substantially better after noise addition (0.457 vs 16.06)	direct PSLG-RF comparison used a synthetic benchmark and synthetic noise rather than an independent experimental MRI dataset	[2]
magnetic descriptors: *H*_c_, *M*_r_, and *M*_s_	gradient boosting, LightGBM, XGBoost, ExtraTrees, random forest, ElasticNet	1,758 total records; *H*_c_ = 1,298, *M*_r_ = 1,298, *M*_s_ = 460	80:20 train and test split with fivefold CV and grid-search optimisation	*R*^2^, RMSE	*H*_c_: gradient boosting and XGBoost, *R*^2^ = 0.78; *M*_r_: gradient boosting, *R*^2^ = 0.85; *M*_s_: ExtraTrees, *R*^2^ = 0.93	dataset size differed substantially among endpoints, particularly for *M*_s_; evaluation remained within the curated source dataset	[9]
dynamic nanoparticle toxicity, including Fe_3_O_4_	ExtraTrees, LightGBM, random forest, XGBoost regressors	2,664 records for SiO_2_, ZnO, and Fe_3_O_4_	80:20 train and test split; subsequent Optuna tuning of the selected LightGBM model	*R*^2^, RMSE	ExtraTrees gave the strongest initial matched comparison (*R*^2^ = 0.9994, RMSE = 1.3193); LightGBM was subsequently selected and optimised	dataset covered only three nanoparticle types and an *E. coli* bioluminescence endpoint; performance cannot be assumed to represent mammalian SPION toxicity	[15]

These within-study comparisons show that no single AI/ML method consistently outperformed the alternatives across magnetic nanoparticle applications. For SAR prediction, ANN and tree-based ensembles performed better than SVR, KNN, and a single decision tree in a heterogeneous literature-derived dataset [29]. When temperature evolution was modelled with an explicit heat-balance constraint, the PINN outperformed both ANN and random forest [30]. In the smaller synthesis dataset, SVR produced the lowest prediction errors [44], whereas tree ensembles performed best for magnetic descriptors, with the preferred ensemble differing among *H*_c_, *M*_r_, and *M*_s_ [9]. MRI signal modelling was condition-dependent: Random forest performed better on clean synthetic data, whereas PSLG was substantially more resistant to imposed noise [2]. Ensemble methods also performed strongly for dynamic toxicity prediction [15]. Overall, relative performance depended on the endpoint, dataset size and structure, descriptor representation, noise, preprocessing, and validation design. Algorithm performance should therefore be interpreted from matched within-study comparisons rather than numerical rankings across unrelated studies.

Several weaknesses recurred. Many models used small, local datasets with a large ratio of candidate variables to observations. Complex ANN architectures can fit such datasets well but may remain sensitive to split selection and hyperparameter choice. Independent external validation across new laboratories, particle batches, scanners, biological models, or treatment settings was rare. Uncertainty was usually reported as point error metrics rather than prediction intervals or calibrated confidence estimates. Code, preprocessing workflows, and complete datasets were not consistently available.

Models trained on simulated data require validation against independent experimental measurements, not only against further outputs from the same simulator. The reinforcement-learning SPION sensor study experimentally validated selected particle diameters [7]. SPIONs from 5 to 15 nm were synthesised at 2 nm intervals, confirming 9 nm as optimal for calcium sensing and 11 nm as optimal for dopamine sensing. Agreement between predicted and experimental optima directly validated the automated design search. Shirmahd et al. combined experimental and simulated data [32]. This strategy produced an overall correlation coefficient of 0.98861 and a test-set coefficient of 0.98641. Transport and flow surrogates require more cautious interpretation when validation remains within the numerical model that generated the training data [61–63]. Akhtar et al. trained a two-input, ten-hidden-neuron, one-output Levenberg–Marquardt network on data generated by its cardiovascular transport model [61]. They also used a ten-neuron hidden layer and a 70:30 training-to-validation split for numerically generated pulsatile flow data [62]. Alsaab et al. used approximately 17,000 CFD-derived velocity points and reported *R*^2^ = 0.99088 for KNN, while explicitly noting the absence of direct experimental or in vivo validation [63]. These results indicate fidelity to numerical generators rather than validated prediction of in vivo transport.

Table 3 consolidates the descriptor-level evidence across the reviewed AI/ML studies and shows how different descriptor classes shaped model outputs, interpretation, and validation requirements.

**Table 3:** Descriptor classes and validation implications in AI/ML studies of MRI-visible magnetic nanocarriers. The table maps the main descriptor classes used across the reviewed literature to their modelling roles, linked outputs, interpretive limitations, and validation requirements.

Descriptor class	Model-visible information	Represented variables	Modelling role	Outputs	Main limitation	Validation requirement	Ref.

stability, surface, and exposure descriptors	conditioned particle state in biological or dispersion media	hydrodynamic size, PDI, zeta potential, coating, charge, medium conditions, concentration, dose, exposure time	prediction of stability, aggregation, uptake, and toxicity	viability, uptake, toxicity class, zeta potential, membrane interaction, aggregation propensity	meaning varies with medium, cell type, and exposure conditions	requires validation in relevant media and exposure conditions	[4,18,24]
structural and core descriptors	core structure and composition underlying magnetic behaviour	core type, size, crystallinity, morphology, composition, architecture, and geometry	connected material structure with magnetic, relaxivity, synthesis, and safety-related outputs	*M*_s_, *M*_r_, *H*_c_, particle size, SAR, *T*_2_ relaxivity, viability classification	core descriptors alone do not capture coating effects, dispersion state, aggregation, biological exposure, or imaging protocol	models require testing beyond the original material family and particle-size range	[4,16,40–42]
magnetic and field descriptors	the magnetic response of the carrier under externally imposed field conditions	magnetisation, coercivity, remanence, anisotropy, exchange bias, concentration, and field/AMF conditions	prediction of MRI response, heating, magnetic coupling, and actuation	SAR, temperature rise, *r*_1_/*r*_2_ relaxivity, 1/*T*_2_, exchange bias, magnetic motion	depends on coating, clustering, water access, concentration, localisation, and acquisition conditions	validation should separate intrinsic magnetic predictability from system-level MRI or delivery performance	[2,7,41,43–45,47–48]
process and formulation descriptors	experimentally adjustable synthesis and formulation variables	process settings, formulation ratios, particle content, coating conditions, and drug loading	optimisation of controllable formulation inputs and material-performance outputs	particle size, relaxivity, magnetic response, SAR, swelling, porosity, mechanics, and release kinetics.	high performance may describe a local formulation space rather than a transferable design rule	validation in new batches, synthesis conditions, and formulation ranges is needed	[15,45–48]
drug, cargo, and therapeutic descriptors	the therapeutic component and its relationship with carrier composition or stimulus conditions	drug type, loading, release conditions, stimulus, formulation, and targeting component	prediction of loading, release, targeting, cytotoxicity, and therapeutic response	drug loading, release kinetics, IC_50_, uptake, targeting response, therapeutic response	release or cytotoxicity alone does not confirm MRI visibility, tumour delivery, or systemic safety	multi-output validation is needed so release, imaging, stability, and biological response are evaluated together	[11–12,49,51]
imaging and acquisition descriptors	how magnetic nanocarrier signal was converted into quantitative imaging readouts	relaxation metrics, MRI/MPI signal features, SPION maps, total iron, and segmentation-derived features	quantified SPION burden, labelled-cell distribution, graft retention, probe accumulation, or response-associated iron uptake	SPION burden, labelled-cell maps, graft signal, tumour accumulation, and response prediction	acquisition dependence limits transfer across scanners, sequences, field strengths, segmentation methods, and signal ranges	requires validation across scanners, protocols, phantoms, biological settings, and reader/segmentation references	[27–30,32–34]
transport, pharmacokinetic, and magnetic-control descriptors	nanocarrier movement, biodistribution, and magnetic guidance under flow or actuation	flow, geometry, particle concentration, field inputs, actuation variables, PK compartments, uptake, and delivery efficiency	prediction of transport, deposition, navigation, localisation, and biodistribution	particle concentration, velocity, trajectory, swarm geometry, tumour delivery efficiency, localisation, retained signal	may reproduce assumed geometry, rheology, and field conditions rather than in vivo tumour delivery	validation against experimental flow systems, biological transport, biodistribution, and in vivo localisation is needed	[60–68]
dataset provenance and validation descriptors	the credibility and transferability of the model evaluation	data provenance, simulation source, validation design, external testing, and reference standard	clarifies whether performance reflects generalisable behaviour or dataset-specific structure	*R*^2^, RMSE, MSE, NMAE, accuracy, F1 score, ICC, cross-validation performance, segmentation agreement	internal validation may be optimistic without separation of related formulations, simulations, or patient-level data	requires validation across independent datasets, batches, scanners, biological models, or patient-level partitions	[3,5,7,10,20,31–32,35–39,61–65]

## Conclusion

### Current AI/ML capabilities in magnetic nanocarrier development

AI is a useful methodological layer in the development of MRI-visible magnetic nanocarriers, but not yet an integrated design framework for clinically translatable cancer drug delivery. Its strongest applications were bounded tasks, including toxicity or uptake modelling from exposure and surface descriptors and zeta-potential prediction from environmental and structural variables [4], magnetic descriptor and relaxivity modelling from core, coating, concentration, and field inputs [9,32], image-derived SPION quantification [3,5,31,35–39], and optimisation of heating, synthesis, release, or magnetic control within constrained experimental or simulated domains [39,42–44,61–66]. These applications show that AI can reduce trial-and-error and expose nonlinear relationships that one-variable strategies miss in well-sampled domains [2,6,10]. In small toxicity and transport datasets, apparent nonlinearity may reflect dataset-specific structure, so this claim should be limited to adequately sampled problems [8,16–17]. The evidence does not yet show that AI can jointly optimise all functions required of an MRI-visible therapeutic nanocarrier.

Direct quantitative comparisons between AI-assisted design and conventional experimental optimisation were uncommon. Where comparisons were possible, the advantage of AI was primarily in navigating multivariable design spaces rather than consistently improving every material endpoint. Reinforcement-learning optimisation identified a 9 nm SPION calcium sensor with a reaction rate of 0.156 s^−1^ and half-life of 4.44 s, compared with 0.066 s^−1^ and 10.5 s for the previous 12 nm design [7]. Conversely, GRA-APSO-ANN optimisation reduced α-Fe_2_O_3_ particle size from 13.7 to 8.5 nm, but *T*_2_ relaxivity decreased from 36.4 to 34.8 mM^−1^·s^−1^ [43]. Thus, AI can accelerate multivariable optimisation, but evidence that it consistently outperforms conventional experimental optimisation remains limited because studies rarely report matched comparisons in experiment number, time, cost, or final performance.

The most consistent finding was that magnetic nanocarrier performance depends on descriptor interactions rather than isolated variables. Structural descriptors defined core architecture and particle dimensions. Surface descriptors captured coating chemistry, charge, and colloidal state. Environmental descriptors represented pH, ionic strength, concentration, and exposure duration. Magnetic descriptors encoded core type and field responsiveness, while acquisition descriptors determined how these properties were translated into MRI or image-derived outputs [10,14–16,21–22,31,38,50]. The biologically active nanoparticle is therefore not the as-synthesised core alone, but the dispersed, coated, possibly aggregated, and conditioned nanoplatform encountered by cells, tissues, and imaging systems. Toxicity and uptake models support this interpretation [8,15–19]. The stability evidence provides the physicochemical basis: Electrostatic repulsion depends on pH and ionic strength, polymer coatings add steric or electrosteric barriers, and magnetic interactions may promote collective behaviour when stabilisation is insufficient. The conditional nature of descriptors was evident across studies. Uptake was predicted better in endothelial than pancreatic cells from identical descriptors (*R*^2^ = 0.885 vs 0.822) [17]. The maghemite toxicity transition occurred near 15 nm, whereas peak predicted viability occurred at 67.5 nm hydrodynamic diameter [16,18]. Chitosan coating reduced SAR by 42% but improved biocompatibility elsewhere [8,45]. Size therefore has different meanings depending on whether the measured response is magnetic performance, cytotoxicity, or hydrodynamic behaviour. AI adds value when descriptors encode the real particle state, but learned importance remains bound to medium, cell type, and measured response.

A similar distinction applies to MRI and magnetic performance. *M*_s_, *H*_c_, and *M*_r_ form an intermediate layer between core structure and imaging response, but they do not alone determine relaxivity or quantitative signal behaviour [16,19–21]. Relaxivity and image contrast depend on size, magnetisation, concentration, field strength, coating architecture, water accessibility, clustering, and acquisition protocol. This asymmetry is mechanistic, not a model artefact. SAR and intrinsic descriptors depend on core composition, size relative to the superparamagnetic threshold, and the Néel–Brownian balance under specified AMF amplitude and frequency. A physics-informed network reproduced this comparatively structured descriptor space with high internal accuracy (*R*^2^ = 0.98). Within the same magnetic-hyperthermia dataset, the physics-informed neural network also showed stronger predictive performance than the purely data-driven models, with *R*^2^ = 0.98 and RMSE = 0.61, compared with *R*^2^ = 0.96 and RMSE = 1.10 for ANN and *R*^2^ = 0.93 and RMSE = 1.42 for random forest. This matched comparison indicates that incorporation of the heat-balance constraint improved prediction of temperature-time profiles under the evaluated conditions. However, the comparison is limited to magnetic hyperthermia and does not establish general superiority of physics-informed models across other magnetic nanocarrier applications. Relaxivity is less directly tied to core magnetism because it also depends on clustering, water accessibility, coating architecture, concentration, and acquisition protocol. Particles with similar magnetic cores can therefore produce different *r*_2_ values under different measurement conditions. Image analysis addressed a different problem by converting SPION-induced signal effects into estimates of labelled-cell burden, graft retention, probe accumulation, or treatment-response metrics [32–35,38]. AI therefore shifts MRI-visible nanocarriers from qualitative contrast agents toward quantitative theranostic readouts.

AI has matured through specialised tasks addressing different layers of magnetic nanocarrier performance. Models of heating, synthesis, release, imaging, transport, and magnetic navigation showed how formulation, field conditions, image features, or flow environments shape nanocarrier behaviour [46,48,61–66]. Their value lies in resolving design constraints that would otherwise require extensive empirical testing. The limitation is that their outputs are rarely connected within the same optimisation framework. A formulation optimised for magnetic heating may not be optimal for MRI contrast, colloidal stability, tumour accumulation, or controlled release. The field therefore needs multiobjective models balancing stability, MRI visibility, magnetic responsiveness, drug loading, release, biodistribution, safety, and therapeutic effect.

### Multiobjective optimisation of magnetic nanocarriers

The Results section showed that most magnetic nanocarrier studies optimised a single dominant endpoint, while the few integrated approaches remained weakly coupled or implicitly multitarget rather than explicitly multiobjective. Multiobjective optimisation could address this limitation by linking common design variables, including core composition and size, coating chemistry, drug loading, concentration, and field conditions, to several performance endpoints and identifying Pareto-optimal formulations rather than a single optimum. Pareto-based methods such as non-dominated sorting genetic algorithm II (NSGA-II) are suited to this problem because they retain solutions representing different trade-offs among competing objectives. In non-magnetic nanocarriers, Abu Sharar et al. used NSGA-II to optimise particle size, zeta potential, and drug-loading efficiency in fluphenazine nanocomposites [71]. Multiobjective Bayesian optimisation provides an alternative when experimental measurements are costly, because a surrogate model guides successive experiments toward informative regions of the design space. Mottafegh et al. applied this approach to the autonomous manufacture of doxorubicin-loaded liposomes and polymeric nanoparticles, jointly targeting particle size and drug loading [72]. Rahdar and Fathi-karkan similarly used multiobjective Bayesian optimisation to minimise normal-cell cytotoxicity while maximising drug loading in curcumin nanocarriers [73]. These strategies could be adapted to magnetic nanocarriers by jointly considering relaxivity, magnetic heating or responsiveness, colloidal stability, drug loading and release, and toxicity. The resulting Pareto front would identify formulations in which improvement of one function requires compromise in another, making these trade-offs explicit. Selected Pareto-optimal formulations would then require experimental validation across the relevant imaging, magnetic, delivery, and biological endpoints.

### Translation and generalisability of AI/ML models

The included studies support a precise conclusion: AI improves prediction and image-based quantification when the material system, imaging protocol, flow geometry, or biological model is well defined. In these settings, models can capture relationships that are difficult to resolve experimentally, including the dependence of transport on field conditions and the effect of magnetic actuation on particle motion. Delivery modelling becomes meaningful when flow, magnetic force, and particle motion are treated as coupled variables [61,63–66]. However, magnetic guidance in a simplified vessel or actuation system does not reproduce all conditions that determine tumour accumulation. In vivo delivery also depends on vascular permeability, interstitial pressure, immune clearance, margination, tissue penetration, and patient-specific anatomy. These models clarify field-particle control, but their predictions require biological validation before they can be interpreted as tumour-delivery evidence. Ferumoxytol-enhanced MRI is one of the few clinically oriented examples in which iron-oxide uptake was evaluated as an imaging biomarker of therapeutic response. Its interpretation remains constrained by validation structure: The reported accuracies of 74% for thoracoabdominal lesions and 79% for brain metastases were derived from 49 thoracoabdominal metastases in 19 participants and 19 brain metastases in seven participants. When multiple lesions are analysed from the same patient, lesion-level validation may overestimate independence unless participant stratified. These findings provide early clinical evidence for response-associated ferumoxytol uptake, but not clinical readiness without broader patient-level validation.

Clinical translation requires evidence at both the nanomaterial and AI/ML levels. Ferumoxytol illustrates the first requirement. Diagnostic ferumoxytol MRI was historically performed outside its therapeutic indication, making indication-specific safety and efficacy evidence necessary. In a multicentre registry of 3,215 patients receiving 4,240 diagnostic ferumoxytol administrations, no severe, life-threatening, or fatal adverse events were reported, although mild and moderate reactions occurred [74]. This supports the feasibility of clinical MRI use but does not establish the validity of a specific imaging biomarker or AI-derived clinical decision. For the AI/ML component, regulatory evaluation requires evidence that model performance is reliable for its intended clinical use. Analyses of approved AI/ML medical devices have identified substantial variation in regulatory evaluation and limited transparency regarding the evidence supporting some devices [75]. Clinical validation should therefore progress from internal testing to independent patient-level validation in representative populations and, ultimately, prospective evaluation of clinical performance and impact. An analysis of AI systems evaluated in randomised clinical trials found considerable heterogeneity in development, external validation, and clinical evaluation pathways, reinforcing the need for stronger external validation before deployment [76]. For magnetic nanocarriers, this gap is particularly important because most current evidence derives from physicochemical experiments, simulations, cell or animal models, or small retrospective and exploratory imaging datasets. Translation to patients will require reproducible manufacturing and safety data together with validation across independent centres, scanners, acquisition protocols, and patient populations before AI-derived predictions can support clinical decisions.

Computational feasibility is another requirement for clinical translation. Most reviewed studies prioritised predictive performance, while computational cost, model size, inference latency, memory demand, and hardware requirements were rarely reported. These factors become important when models must process large imaging datasets or support real-time magnetic navigation and clinical decision-making. More complex architectures may improve accuracy but can reduce deployment efficiency, whereas simpler models or surrogate approaches may provide adequate performance with lower computational demand. Future studies should therefore report inference time and resource requirements alongside predictive metrics and evaluate whether performance is maintained when models are scaled to clinically relevant data volumes and deployment hardware.

Batch-to-batch variability is an additional source of model domain shift. Small changes in synthesis or processing can alter particle size distribution, crystallinity, coating density, surface charge, magnetic properties, and drug loading, even when nominal formulation conditions are unchanged. The reviewed studies show that drying temperature, synthesis conditions, coating composition, and formulation variables can substantially change magnetic and structural outputs [32,43,45]. A model trained on a single production batch may therefore learn batch-specific correlations that do not persist in independently prepared material. This risk is difficult to quantify from the current literature because batch identity and between-batch variability were rarely reported explicitly. Future studies should treat independently synthesised batches as separate experimental units, report between-batch variation in key descriptors, and reserve complete batches for external testing rather than randomly distributing measurements from the same batch across training and test sets. Such batch-level validation is necessary to determine whether model performance reflects transferable structure–property relationships or only reproducibility within a specific manufacturing run.

### Validation requirements and future research priorities

Validation quality remains the main methodological constraint. Some studies used cross-validation, held-out testing, independent validation, expert segmentation comparison, or experimental confirmation of selected optima [16,20,33–34]. External validation across independent laboratories, particle batches, scanners, biological models, and patient cohorts was rare. Small datasets and flexible models risk optimistic estimates. Two patterns illustrate this problem. *M*_s_ was modelled from 460 records yet scored highest (*R*^2^ = 0.93), whereas *H*_c_ and *M*_r_ used 1,298 records but achieved lower scores; accuracy therefore partly tracks favourable sampling rather than learning alone [9]. Curated databases may also distribute near-identical formulations across cross-validation partitions, making internal performance more optimistic than validation on an independent external dataset [10]. Simulated data should be interpreted according to their role in the modelling workflow. Monte Carlo augmentation of experimental data and synthesis-confirmed optima can strengthen local model development when tied to measured systems [7,32]. By contrast, surrogates trained entirely on CFD outputs can achieve near-perfect internal agreement (*R*^2^ > 0.99) while mainly reproducing numerical-model assumptions rather than biological delivery. The concern is not only overfitting, but learning from a generator whose geometry, rheology, and field homogeneity differ from tumour transport. AI can support preclinical prioritisation by identifying influential descriptors, narrowing formulation search, and improving quantitative imaging readouts. Translation requires stronger evidence of reproducible manufacturing, stability in biological media, long-term biodistribution and clearance, immune and organ safety, validated MRI or MPI protocols, and transparent algorithmic performance under intended-use conditions.

Heterogeneous experimental protocols are a major barrier to cross-study reproducibility. Differences in synthesis conditions, particle characterisation, AMF settings, biological assays, exposure conditions, MRI field strength, pulse sequences, and preprocessing can change both input descriptors and measured outcomes, even for nominally similar magnetic nanomaterials. As a result, models may capture protocol-specific correlations rather than transferable structure–property relationships. Cross-study reproducibility therefore requires harmonised reporting of experimental conditions, protocol-aware descriptor design, and external validation using data generated under different laboratories, instruments, and acquisition settings.

Uncertainty estimation and confidence calibration are also largely absent from the reviewed literature. Most studies report point-performance metrics such as *R*^2^, RMSE, accuracy, or F1 score, which describe average predictive performance but do not indicate how reliable an individual prediction is. This distinction is important in magnetic nanomedicine because predictions may be applied to new particle compositions, formulations, imaging conditions, or biological systems that differ from the training data. A model may retain a high overall accuracy while remaining poorly calibrated, meaning that its reported confidence does not correspond to the actual probability of a correct prediction. Future studies should therefore complement conventional performance metrics with prediction intervals or calibrated confidence estimates and evaluate whether these remain reliable on independent validation data. Such reporting would allow users to distinguish high-confidence predictions within the modelled domain from uncertain extrapolations that require additional experimental confirmation.

Transferability across magnetic nanoparticle compositions, coatings, and nanocarrier architectures remains poorly established. Models trained on compositionally diverse datasets provide some evidence that common descriptors can capture relationships across Fe_3_O_4_, Fe_2_O_3_, CoFe_2_O_4_, and related magnetic materials [9–10], but performance within a pooled dataset does not demonstrate transfer to a previously unseen composition. The same limitation applies to surface chemistry. Coating changes can alter hydrodynamic behaviour, surface charge, biological interactions, magnetic heating, and MRI response [8,17,24,45], so relationships learned for one coating class cannot be assumed to persist after substitution with silica, chitosan, polymeric, or other surface architectures. Transfer across nanocarrier platforms is even less established because ferrogels, scaffolds, nanofibres, free nanoparticles, and hybrid carriers differ in matrix structure, transport behaviour, drug loading, magnetic-particle mobility, and exposure conditions [48–49,51]. Most reviewed models were developed and validated within a single material family or carrier platform rather than tested prospectively on a distinct formulation class. Demonstrating transferability will therefore require validation in which complete compositions, coating chemistries, or carrier architectures are withheld during model development and used only for external testing. Such evaluation would distinguish models that capture transferable structure–property relationships from those that primarily interpolate within the material domain represented in the training data.

The next step is to test whether model behaviour survives contextual change. A prediction based on one formulation, scanner, or biological model has limited value unless the same descriptor logic remains valid under new experimental conditions. This requires validation in new materials and measurement settings, not only another internal train-test split. Multiobjective modelling will be meaningful only when improvements in one function, such as relaxivity or release, are tested against the same formulation’s stability, safety, biodistribution, and therapeutic effect. Model interpretation can identify variables worth testing, but it should not be treated as mechanistic evidence on its own. A stronger approach is to let the model identify a plausible design variable, alter it experimentally, and test whether stability, imaging response, and biological behaviour change as expected.

A standardized benchmarking framework would improve comparison across diverse magnetic nanomedicine applications. Benchmarking should use clearly defined endpoint-specific datasets, fixed training-validation-test partitions, common baseline models, and agreed performance metrics appropriate to each task, such as *R*^2^ and RMSE for regression or accuracy, F1 score, and AUROC for classification. Reporting should also include uncertainty and calibration, computational cost, dataset provenance, experimental conditions, and external-test performance. Where possible, benchmark sets should include independent laboratories, particle batches, scanners, and biological systems. Such a framework would allow model performance to be compared reproducibly within application domains while avoiding inappropriate ranking across fundamentally different endpoints.

For researchers planning AI/ML studies in this field, the evidence supports several methodological priorities. Models should be designed around a clearly defined intended endpoint and should include descriptors that represent the experimentally relevant particle state, including core properties, coating, hydrodynamic behaviour, concentration, exposure conditions, and, where applicable, magnetic-field and imaging parameters. Data splitting should preserve independence at the level that matters experimentally; particle batches, laboratories, scanners, animals, or patients should not be distributed across training and test sets when this would create information leakage. Studies should report dataset size, feature dimensionality, missing-data handling, preprocessing, hyperparameter optimisation, uncertainty, calibration, and performance against simple baseline models. External validation should then test new particle batches, material compositions, biological systems, scanners, or patient cohorts, followed where possible by prospective experimental confirmation of model-selected formulations. Specific research priorities are therefore the development of shared benchmark datasets, multiobjective models that integrate imaging, magnetic, delivery, and safety endpoints, physics-informed or mechanistically constrained models for data-limited settings, formal uncertainty quantification, and prospective cross-laboratory and clinical validation. These steps would shift the field from high-performing task-specific models toward reproducible AI/ML frameworks capable of supporting magnetic nanocarrier design and translation.

## Data Availability

Data sharing is not applicable as no new data was generated or analyzed in this study.
